# What is the effect of intergenerational activities on the wellbeing and mental health of older people?: A systematic review

**DOI:** 10.1002/cl2.1355

**Published:** 2023-10-03

**Authors:** Rebecca Whear, Fiona Campbell, Morwenna Rogers, Anthea Sutton, Ellie Robinson‐Carter, Richard Sharpe, Stuart Cohen, Ronald Fergy, Ruth Garside, Dylan Kneale, G. J. Melendez‐Torres, Joanna Thompson‐Coon

**Affiliations:** ^1^ NIHR ARC South West Peninsula (PenARC) University of Exeter Medical School University of Exeter Exeter UK; ^2^ School of Health and Related Research The University of Sheffield Sheffield UK; ^3^ School of Health and Related Research University of Sheffield Sheffield UK; ^4^ Co‐Chair “Only Connect!” Network & Conference Series "Only Connect!" Truro UK; ^5^ Public Health Cornwall Council & University of Exeter Medical School St. Austell UK; ^6^ NHS Kernow Clinical Commissioning Group St. Austell UK; ^7^ London UK; ^8^ European Centre for Environment and Human Health, University of Exeter Medical School University of Exeter Truro UK; ^9^ EPPI‐Centre, Social Science Research Unit University College London London UK; ^10^ Peninsula Technology Assessment Group University of Exeter Medical School Exeter UK

## Abstract

**Background:**

Opportunities for social connection between generations have diminished over the last few decades around the world as a result of changes in the way that we live and work. The COVID‐19 pandemic has exacerbated loneliness for many with young and old being kept apart for safety. The Public Health England prevention concordat for better mental health (Office for Health Improvement and Disparities) aims to bring a prevention‐focused approach to improving public mental health. The concordat promotes evidence‐based planning and commissioning to increase the impact on reducing health inequalities using sustainable and cost‐effective interventions that impact on the wider determinants of mental health and wellbeing for children and young people and older people. Intergenerational activities could provide an opportunity to support both populations. In 2023, we produced an evidence and gap map to illustrate the amount and variety of research on intergenerational interventions and the gaps in research that still exist in this area. The review conducted here is based on the evidence in that map.

**Objectives:**

This systematic review examines the impact of intergenerational interventions on the wellbeing and mental health of older people and identifies areas for future research as well as key messages for service commissioners.

**Search Methods:**

We searched an evidence and gap map published in 2022 (comprehensive searches conducted July 2021 and updated June 2023) to identify randomised controlled trials of intergenerational interventions that report mental health and wellbeing outcomes for older people.

**Selection Criteria:**

Randomised controlled trials of intergenerational interventions that involved unrelated younger and older people with at least one skipped generation between them and reported mental health or wellbeing outcomes for older people were included in this review.

**Data Collection and Analysis:**

We used standard methodological procedures expected by The Campbell Collaboration. We conducted data extraction and Cochrane risk of bias assessments in EPPI reviewer. Where data allowed meta‐analyses were conducted in STATA.

**Main Results:**

This review includes 14 trials from six different countries. The trials had some important methodological weaknesses. Interventions were mainly delivered in‐person and often in groups. They included visiting programmes, school volunteering programmes, music‐based interventions and task‐oriented interventions such as activities set in a multigenerational park, reminiscing activities, aggression management programmes, learning a language, making local environmental changes and in‐school project work. Intergenerational interventions showed a small positive trend towards improving self‐esteem (effect size [ES]: 0.33, 95% confidence interval [CI]: −0.35, 1.01) and depression (ES: 0.19, 95% CI: −0.23, 0.60) for older people participating. However, due to the small study sizes and low number of studies available, we cannot be confident about any effects. The results for other mental health and wellbeing outcomes are reported but due to little overlap in similar assessments across the studies, we could not combine them to assess the strength of evidence. There were no data about social isolation, spiritual health or sense of community. There are no long‐term studies and no data on equity. We still know very little about what works and how or why. Whilst some interventions do use theories and logic to inform their development others do not. More exploration of this is needed.

**Authors’ Conclusions:**

Commissioners and intervention developers should ensure interventions provide sufficient theoretical evidence for the logic behind the proposed intervention and should improve their consideration of equity within the interventions Research on intergenerational interventions need more consistent and agreed measures for reporting outcomes including community outcomes (core outcome sets). More understanding is needed on how best to measure ‘community’ outcomes. Research on intergenerational interventions should measure outcomes for BOTH the older and younger population engaged in the intervention—these may or may not be the same outcomes reflected in both populations. Further research is needed on the long‐term impact of interventions on outcomes (whether participants need to keep being involved in an ‘intervention’ to continue to benefit) and sustainability of interventions beyond the initial funding of the research project. Supporting this our stakeholders highlighted that interventions that are initiated for research and then end (usually within a year) are not helpful.

## 1 PLAIN LANGUAGE SUMMARY

### 1.1 There is limited evidence of intergenerational interventions’ effects on mental health and wellbeing of older people

Intergenerational interventions are activities designed to bring younger and older people together, and may contribute to small improvements in self‐esteem and levels of depression in older people.

However, this systematic review shows that it is not clear if these positive effects are consistent or last beyond the intervention. The evidence in this review also suggests that it is not clear if intergenerational interventions have any impact on quality of life, agitation, stress and loneliness in older people. There is no trial evidence looking at the effects of intergenerational interventions on social isolation for older people.

#### 1.1.1 What is this review about?

Mental health and wellbeing, including loneliness, is a huge global issue, shared by younger and older people. The Covid‐19 pandemic has increased loneliness for many, with generations being kept apart for safety.

Intergenerational interventions aim to promote greater understanding and respect between generations and help build communities. Intergenerational interventions can take many forms: school children visiting nursing home residents to share activities and stories, younger and older people coming together to share in music‐based activities, older people volunteering in schools, and older people from outside the family helping/mentoring students.

This review looks at the impacts of intergenerational interventions related to the mental health and wellbeing of older people including depression, anxiety, quality of life, self‐esteem, social isolation and loneliness. The review also looks at impacts on life satisfaction, agency (a sense of control and desire to do things in life), generativity (sense of purpose/meaning in life), happiness, intergenerational interaction or interaction with others, social activities, self perception, perceived emotional wellbeing, spiritual health, and sense of community.

#### 1.1.2 What is the aim of this review?

This Campbell systematic review describes trials of intergenerational interventions that have reported on the mental health and wellbeing of older people, and how effective they were.

#### 1.1.3 What studies are included?

This review includes 14 trials from six countries: USA, Japan, Italy, Spain, Australia and Canada. Interventions were mainly delivered in person and often in groups. They included visiting programmes, school volunteering programmes, music based interventions and task‐oriented interventions such as activities set in a multigenerational park, reminiscing activities, aggression management programmes, learning a language, making local environmental changes and in‐school project work.

The trials had some important weaknesses that may have affected their results.

#### 1.1.4 What are the main findings of this review?

Intergenerational interventions showed a small positive trend towards improving self‐esteem and depression for older people participating. However, due to the small study sizes and low number of studies available, we cannot be confident about any effects.

Results for other mental health and wellbeing outcomes are reported. There were no data about social isolation, spiritual health or sense of community.

The lack of consistent outcomes reported and the lack of studies on interventions that are similar or have similar elements means it is difficult to determine if any one intervention or element is effective for any given outcome.

#### 1.1.5 How do these interventions work?

We still know very little about what works and how or why. Whilst some interventions do use known theories or techniques to articulate how something is thought to have an impact, others do not. It is therefore difficult to establish why any particular intervention might have an impact on any particular outcome.

#### 1.1.6 What do the findings of this review mean?

The differences in the included studies means we cannot be certain that the findings are true and consistent across intergenerational activities. We need more robust research with larger numbers of participants who are studied for a longer period and after the intervention.

This field of study would also benefit from using agreed outcome measures consistently across interventions, to aid future comparisons and the development of research and practice.

#### 1.1.7 How up‐to‐date is this review?

The review authors searched for studies up to July 2021 and searched again in June 2023 for new randomised controlled trials.

## 2 BACKGROUND

### 2.1 The problem, condition or issue

Although multigenerational families are reported to be on the increase recently in the US (Generations United, 2021), the number of multigenerational families with intergenerational support varies across rural and metropolitan areas and different cultures (ILC, 2012). In rural settings, intergenerational patterns of socialisation are often disrupted as younger people migrate to cities, missing opportunities to benefit from the knowledge and guidance of older family members. Opportunities for social connection between generations have diminished over the last few decades in the UK as a result of changes in the way that we live and work (Kingman, 2016; United for all Ages, 2017) and around the world Ending (Loneliness, 2022; Van Beek, 2022). Housing and economic trends have seen younger people move to live in city centres whilst the older generation live in towns and rural areas. A report published by the Intergenerational Foundation in 2016 (Kingman, 2016) suggests that in the 25 biggest cities within the UK only 5% of people aged over 65 live in the same neighbourhood as someone under the age of 18. Furthermore, even when people from different age groups do live in the same area, the decline in spaces such as libraries, youth clubs and community centres mean that there are fewer opportunities to meet and mix socially with other generations outside our own families. Increased working hours, improved technology, changes in family patterns, relationship breakdowns within families and migration are also believed to be contributory factors to generation segregation (Generations Working Together, 2019). There are many potential economic, social and political impacts of generations living separate and parallel lives, for example, higher health and social care costs, an undermining of trust between generations (Brown, 2014; Edström, 2018; Laurence, 2016; Vitman, 2013), reduced social capital (Laurence, 2016), a reliance on the media to form understanding of others’ viewpoints (Edström, 2018; Vasil, 1993) and higher levels of anxiety and loneliness. Loneliness is a huge global issue (Surkalim, 2022) and one that is shared by both younger and older people (Hong, 2023). The COVID‐19 pandemic has exacerbated loneliness for many with young and old being kept apart for safety.

In the Office for National Statistics Community Life Survey, 2016 to 2017 (ONS, 2018) 5% of adults in the UK felt lonely (often or always). Similarly, in the US a survey conducted in 2018 found that almost half of 20,000 U.S. adults sometimes or always reported feeling alone with 40% of participants also reporting they sometimes or always feel that their relationships are not meaningful and that they feel isolated (Novotney, 2019). In the UK those aged 16–24 were also more likely than all other age groups (except the 25–34 years group) to report feeling lonely (often or always). Increased social isolation also reduces mental wellbeing (Hawkley, 2015) in older age and is further impacted by the pandemic due to the measures put in place to prevent spread of the virus. This was found to have an adverse impact on psychological outcomes including increased depression and anxiety (Robb, 2020; Zhou, 2020). There are a range of interventions designed to help older people who feel socially isolated and/or lonely including community support groups, visiting schemes, therapy/counselling schemes, and interventions to promote physical activity and other social activities (Dickens, 2011). Intergenerational interventions are one option that can combine social interaction and connection across generations using meaningful and engaged activities which can help to tackle feelings of loneliness and social isolation and improve wellbeing.

### 2.2 The intervention

We use the definition of intergenerational practice developed by the Beth Johnson Foundation:

Intergenerational practice aims to bring people together in purposeful, mutually beneficial activities which promote greater understanding and respect between generations and contributes to building more cohesive communities. Intergenerational practice is inclusive, building on the positive resources that the young and old have to offer each other and those around them (Beth Johnson Foundation, 2021).

Intergenerational programmes and activities may be promising interventions that can address some of the needs of both older people and children and young people. These interventions can take many formats and are delivered in diverse settings, often by third sector organisations. Although, evidence suggests that intergenerational activity can have a positive impact on participants (e.g., reducing loneliness and exclusion—for both older people and children and young people; improving mental health; increasing mutual understanding and tackling important issues such as ageism, housing and care) (Canedo‐García, 2017), decisions to commission/fund any services are complex due to the lack of evidence regarding which programmes to fund and support.

Between July and December 2021, we produced an evidence and gap map (EGM) (Campbell Whear, 2023) to illustrate the amount and variety of research on intergenerational interventions and the gaps in research that still exist in this area. We have discussed the evidence from this map with our stakeholders and co‐developed the research question for this review as an important question with both current and future relevance for ageing communities.

### 2.3 How the intervention might work

**Figure 1:**
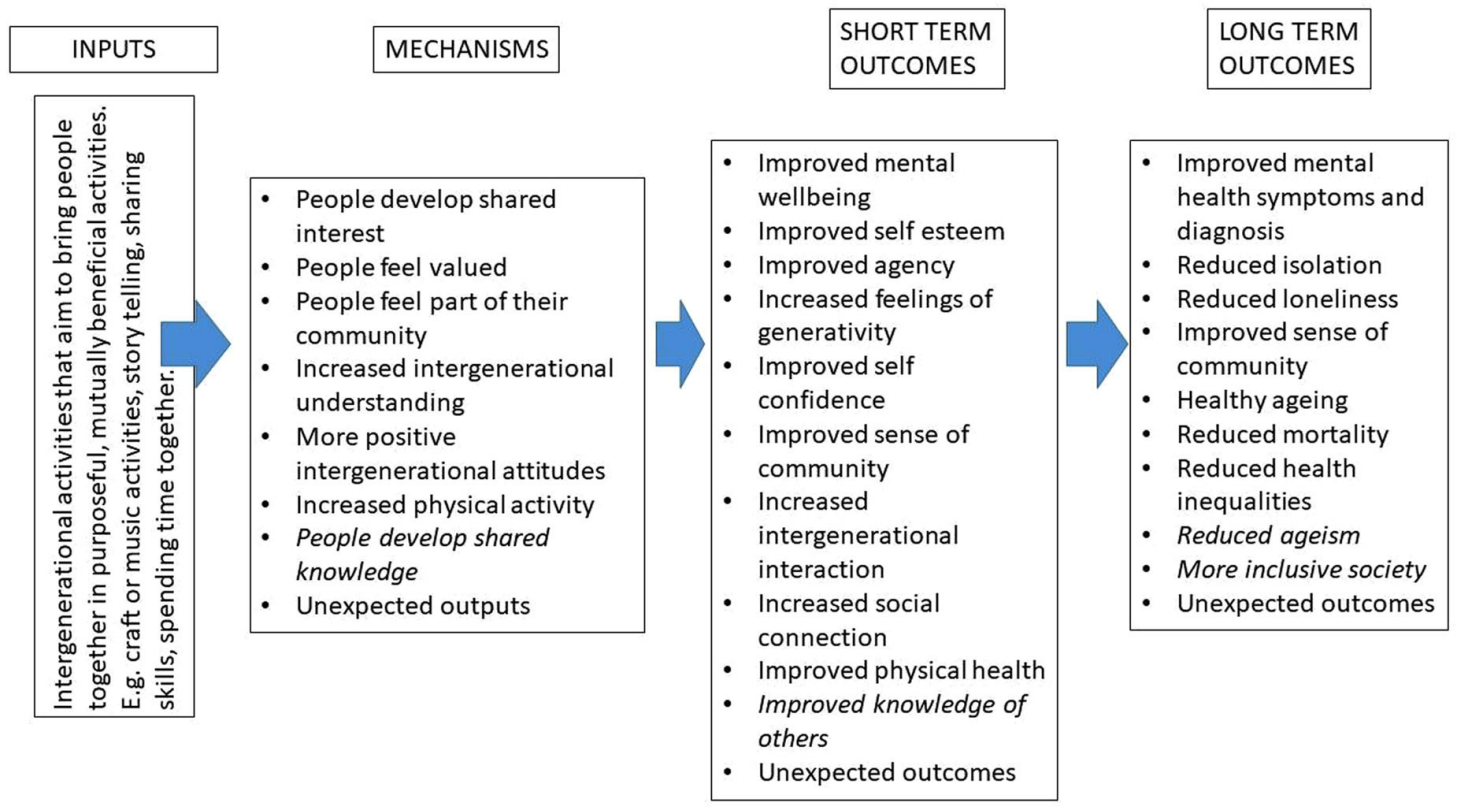
Logic model to illustrate how intergenerational activities might work to improve the mental health and wellbeing of older people.

We have developed a logic model (Figure 1) to illustrate our understanding of how intergenerational activities might work to improve the mental health and wellbeing of older people. The logic model is based on discussions with the stakeholder group during the construction of the EGM (Campbell Whear, 2023). Ronzi (2018) describes evidence for numerous mediators involved in the mechanisms of intergenerational interventions for example activities such as reading to children, assisting young people in school and mentoring activities lead to older people feeling appreciated, valued and respected and being able to share an interest with others. This then leads to more positive attitudes towards ageing, improved self‐esteem and confidence, happiness, enjoyment and satisfaction, which then encourages more social participation, increased social relationships, increased physical activity and decreased social isolation. Vieira (2016) suggests intergenerational practices could be divided into three main types: 1—those that bring generations together to promote intergenerational relationships (focused on solving the problem of the gap between generations); 2—those that combine the promotion of intergenerational relationships with an additional goal, such as, helping older people develop suitable IT skills; and 3—those that bring generations together because it seems a better way to achieve a secondary goal, such as local environment community projects.

The logic model indicates some of the ways that intergenerational activities (in their broader description/context) might work (mechanisms) to impact on various mental health and wellbeing outcome in the short and longer term. There are many areas that are not yet explored or evidenced, and we expect our review to help improve this knowledge.

### 2.4 Why it is important to do this review

The UK's All Party Parliamentary Group on Social Integration—Healing the generational divide report (APPGSI, 2019) offers a range of recommendations to alleviate the generational divide and over 50 voluntary organisations working with MIND (MIND, 2020) advocate for communities, organisations, agencies and the government to work together to respond to the mental health and wellbeing needs of the nation. Evidence‐based intergenerational interventions may have a substantial role to play in this (Dickens, 2011).

It is not just the UK that has identified loneliness and social isolation as a major health risk. In May 2023 the US SurgeonGeneral released Social Connection—Current Priorities of the US Surgeon General (hhs.gov) identifying an ‘epidemic of loneliness and isolation’ that can cause physiological harms, including a 29% increased risk of heart disease; a 32% increased risk of stroke; and a 50% increased risk of developing dementia for older adults as well as those associated with mental health and wellbeing. In Australia 37% of 18–24 year olds are reported to feel lonely as well as one‐third of adults aged over 60 years (Groundswell, 2022). The WHO/UN Decade of Health Ageing report (WHO's work on the UN Decade of Healthy Ageing [2021–2030]) also highlights the need to change how we think, feel and act towards age and ageing, and develop communities in ways that foster the abilities of older people—intergenerational interventions may be a place for both these things to happen.

Having conducted an EGM on intergenerational interventions we were able to identify areas where reviews have and have not already been conducted and areas where research was more or less prolific. We have identified reviews registered on PROSPERO that cover related areas such as meaningful engagement between adolescents and older people in a residential care setting (Laging, 2020) the design and best practice for intergenerational exchange programmes also between adolescents and older people (Webster, 2019) and features of intergenerational programmes and attitude changes between adolescents and older people (Ahmad, 2021).

We have been careful to ensure that our review does not duplicate existing reviews. There is some overlap with a recently published review (Krzeczkowska, 2021) on the effectiveness of intergenerational interventions, although this review included a wide range of study designs and reported on a wider range of outcomes (social, cognitive and health).

Our review includes only randomised controlled trials and is limited to mental health and wellbeing outcomes for older people. However, as our literature search was more comprehensive, we were able to identify a larger body of relevant evidence from randomised controlled trials. Furthermore, in response to stakeholder feedback, we explore the characteristics of intergenerational activities (e.g., type of activity, level of contact, setting, duration) as well as the theories underlying them to gain an understanding of the characteristics associated with a positive outcome for older people.

## 3 OBJECTIVES

This systematic review examines the impact of intergenerational interventions on the wellbeing and mental health of older people and identifies areas for future research as well as key messages for service commissioners.

We seek to answer the following research questions from randomised controlled trial studies:

1. What is the effect of intergenerational interventions on the wellbeing and mental health of older people?
2. What characteristics of intergenerational activities are associated with an impact on the wellbeing and mental health of older people?
3. What are the underlying theories for the effectiveness of intergenerational activities in older people?

## 4 METHODS

### 4.1 Criteria for considering studies for this review

#### 4.1.1 Types of studies

We included randomised control trials (RCTs) only as we wished to understand the effectiveness of these interventions. Control/comparator groups were usual care/no intervention, wait‐list control or intervention but without the intergenerational component. We acknowledge that there is a wider array of intervention designs that can inform our knowledge about these interventions, but randomised trials are possible in this context, and so we wanted to understand the level of knowledge gained from these trials to date to more appropriately inform areas for future research and practice.

#### 4.1.2 Types of participants

We included studies that include older adults and children and young people but were particularly interested in outcomes related to older people.

No age boundary restrictions were applied, but we sought information from studies that suggested there was at least one skipped generation between older and younger participants.  Studies in which participants are related by family or marriage were excluded.  Inclusion was not be determined by age cut‐offs but by the included studies’ own definition of ‘older people’ and ‘younger people’.  The participants of these studies did not have to have reported feelings of loneliness or social isolation.

#### 4.1.3 Types of interventions

We included any intervention that sought to bring older and younger people together intentionally with the purpose of achieving positive health and/or social and/or educational outcomes.  These might include reminiscence programmes, buddy systems, storytelling, school‐based interventions, arts‐based interventions and digital interventions.

We used the Depth of Intergenerational Engagement Scale (Kaplan, 2004) as the framework for the interventions. The Depth of Intergenerational Engagement Scale places programmes and activities on a continuum, with points that correspond to different levels of intergenerational engagement, ranging from initiatives that provide no direct contact between age groups (point 1) to those that promote intensive contact and ongoing opportunities for intimacy (point 7). Examples of intergenerational initiatives fitting into each point on the scale are described below (Table 1).

**Table 1:** Depth of Intergenerational Engagement Scale (Kaplan, 2004).

Level	Description	Example
1	Learning about other age groups. Participants learn about the lives of persons in other age groups, although there is no direct or indirect contact.	‘Learning about Aging’ programs designed to teach youth about aspect(s) of the aging process.
2	Seeing the other age group at a distance. These initiatives facilitate an indirect exchange between individuals of two or more age groups. Participants might exchange videos, write letters, or share artwork with each other, but never actually meet in person	A pen‐pal program in which youth in an after‐school club exchange letters with residents of a nursing home.
3	Meeting each other. Initiatives culminate in a meeting between the young participants and older adults, generally planned as a one‐time experience.	A class of students plan for and visit a local senior centre in which all engage in activities during a July 4th picnic.
4	Annual or periodic activities. Often tied to established community events or organisational celebrations, intergenerational activities occur on a regular basis. Although infrequent, these activities might symbolise intergenerational and community unity and influence attitudes and openness towards additional or ongoing activities.	Intergenerational activities at a school on Grandparent's Day, an annual community dance in which youth and older adults are actively involved, and Christmas carolling at assisted‐living homes.
5	Demonstration projects. Usually involve ongoing intergenerational activities over a defined period of time. Depending on project goals and objectives, the intergenerational exchange and learning can be quite intensive. These initiatives are often implemented on an experimental or trial basis, and frequently depend on external funding.	A 6‐month pilot program, sponsored by an agency that provides teen parenthood support services. Senior adults who have successfully raised children are enlisted to mentor and provide support for pregnant and parenting teens.
6	Ongoing intergenerational programs. Programs from the previous category that have been deemed successful and valuable from the perspective of the participating organisations and the clientele are incorporated as an integral part of their operation. This extends to program and staff development such as preparing individuals to work with populations of various age groups	Based on a partnership forged between a senior centre, a community youth centre, and an environmental education centre, senior adults and youth plan and execute the town's environmental improvement campaign. Systems are established to organise numerous projects, train and assign participants, and provide continuing support and recognition.
7	Ongoing, natural intergenerational sharing, support, and communication. There are times when the intergenerational reconnection theme transcends a distinct program or intervention. This is evident when the social norms, institutional policies and priorities of a particular site, community, or society reflect values of intergenerational reciprocity and interdependence. Intergenerational engagement takes place as a function of the way community settings are planned and established. In this context, opportunities for meaningful intergenerational engagement are abundant and embedded in local tradition	A YMCA facility houses a senior citizen centre. Older adults and youth participate in a variety of age‐integrated activities.

Programs fitting into all points on this continuum provide positive experiences for interacting with persons in other age groups. However, for this project interventions in levels 1 and 2 are outside the scope of our review due to the lack of direct interaction between the generations, all other levels are included.

#### 4.1.4 Types of outcome measures

Only studies that include at least one type of outcome relating to mental health or wellbeing in older people will be included.

##### Primary outcomes

To address Research Question 1 (What is the effect of intergenerational interventions on the wellbeing and mental health of older people?) our primary outcomes included all outcomes reported using a standardised measure (a measure with reported/known reliability and validity) to assess mental health and wellbeing such as depression, anxiety, quality of life, self‐esteem, social isolation and loneliness.

##### Secondary outcomes

To address Research Question 1 our secondary outcomes included other indicators of mental health and wellbeing that are less likely to be captured by standardised measures and more likely to be captured by individual/bespoke questions or observations. For example, reports of life satisfaction, agency, generativity (sense of purpose/meaning in life), happiness, intergenerational interaction/interaction with others, social activities self‐perception, perceived emotional wellbeing, spiritual health, and sense of community.

To address Research Question 2 (What characteristics of intergenerational activities are associated with a positive impact on the wellbeing and mental health of older people?) we used information on intervention characteristics such as setting, context, intensity, duration etc.

To address Research Question 3 (What are the underlying theories for the effectiveness of intergenerational activities in older people?) we used information on the underlying theories reported within the included studies.

#### 4.1.5 Duration of follow‐up

Any duration.

#### 4.1.6 Types of settings

Any setting or context.

#### 4.1.7 Publication status

We did not exclude studies on the basis of publication status.

### 4.2 Search methods for identification of studies

Searches were conducted to populate the EGM (Campbell Whear 2023) from which this review originates. For the map we searched MEDLINE (via OvidSp), EMBASE (via OvidSp), PsycINFO (via OvidSp), CINAHL (via EBSCOHost), Social Policy and Practice (via OvidSp), Health Management Information Consortium (via OvidSp), Ageline (via EBSCOhost), ASSIA (via ProQuest), Social Science Citations Index (via Web of Science), ERIC (via EBSCOhost), Community Care Inform Children, Research in Practice for Children, ChildData (via Social Policy and Practice), the Campbell Library, the Cochrane Database of Systematic Reviews and the CENTRAL database to populate the EGM between 22 July and 30 July 2021 using terms for intergenerational practices. As we were seeking to identify the richest possible evidence base, we did not place any language or date restrictions on the searches.  Our search strategies for the EGM are available in Supporting Information: Appendix 1.

#### 4.2.1 Electronic searches

For the subsequent review in June 2023 we reran the database strategies from the date of the last search for the EGM (July 2021) on the CENTRAL database of randomised controlled trials, and on the databases MEDLINE, PsycINFO, and AgeLine with the addition of a search filter for randomised controlled trials. These databases were selected based on the completion of a search summary table (Bethel, 2021) following the EGM which indicated where relevant studies were found. We also carried out citation searching (forwards and backwards) any included studies.

#### 4.2.2 Searching other resources

For the EGM we also searched for grey literature via relevant organisation websites (Age UK, Age International,  the Centre for Ageing Better, Barnardo's, Children's Commission, UNICEF, Generations Working Together, the Intergenerational Foundation, Linking Generations and The Beth Johnson Foundation), conference abstracts via the Conference Proceedings Citation database, and dissertations via ProQuest Dissertations and Theses Global.  These searches were updated as above.

To find any published literature not captured by the databases we reviewed the included studies within relevant systematic reviews and hand searched the Journal of Intergenerational Relationships.

### 4.3 Data collection and analysis

#### 4.3.1 Selection of studies

Studies were identified from the relevant domains of our EGM (Campbell Whear, 2023) and screened against the eligibility criteria independently by two reviewers. Methods for study selection used to populate the EGM can be found in the report (Campbell Whear, 2023).

#### 4.3.2 Data extraction and management

Once relevant studies were identified data extraction was undertaken by one reviewer and checked by a second with discrepancies resolved by discussion with arbitration by a third reviewer were necessary. Data extraction sheets were developed in EPPI‐Reviewer and piloted by two reviewers on a sample of papers. We extracted the following data: Publication details, sample size, population details—including details required in the PROGRESS Plus criteria (O'Neill, 2014), intervention and comparator details including type of activities undertaken, setting, duration, intensity, timing and mode of delivery—as detailed in the TIDieR checklist (Hoffman, 2014), outcome measures, and outcome data. We also extracted details of the underlying theories and logic as described by the authors in the introduction and method sections of included papers.

#### 4.3.3 Assessment of risk of bias in included studies

One reviewer conducted critical appraisal which was checked by a second, with all discrepancies resolved through discussion. We conducted critical appraisal in EPPI‐Reviewer and used the already incorporated Cochrane Risk of Bias tool (Higgins, 2019).

#### 4.3.4 Assessment of equity in included studies

We used the PROGRESS Plus framework (O'Neill, 2014) to guide data extraction of participant characteristics of eligible and targeted populations within the included studies.

#### 4.3.5 Description of interventions used in included studies

We used the TIDieR checklist (Hoffman, 2014) to describe the interventions used in included studies. The TIDieR checklist contains 12 items that cover the information required to comprehensively describe an intervention and its implementation.

#### 4.3.6 Unit of analysis issues

##### Dealing with missing data

Where data were not available within the published papers, the authors were contacted, and this information was requested. Where authors did not provide the requested information these studies were excluded from the meta analysis but included in the review.

##### Assessment of reporting biases

Too few trials were included in any one meta‐analysis to support use of funnel plots. Reporting biases at outcome level were assessed via inspection of included studies.

##### Data synthesis

We anticipated a disparate and heterogeneous body of evidence in terms of the aim of the intervention, the population, intervention, comparator and outcomes.

We conducted meta‐analysis for outcomes that had three or more contributing studies and followed the Synthesis Without Meta‐analysis (SWiM) reporting guidance for the remaining synthesis (Campbell, 2020). All studies in the meta analyses only reported one measure of each of the outcomes and thus each study only contributed one effect size per meta‐analysis.

Studies are tabulated and grouped according to outcomes, using the logic model to inform decisions on groupings where appropriate. Tables are used to describe the heterogeneity within and across the included studies.

We have used a standard metric (effect size) for each outcome measure where possible. Where meta‐analysis has not been possible, we have used effect size estimates. We have used pre‐reported effect sizes from the studies included in meta analysis using a random effects model Stata.

All outcomes were estimated using standardised mean differences (Cohen's *d*).

Where meta‐analysis was appropriate heterogeneity was described using the *I* ^2^ statistic and the *Q* test. Subgroup analysis could not be performed due to the variation in intervention design. No sensitivity analyses were planned. Given the variation across studies, we used the random effects model. We report the estimate of *χ* ^2^ and the confidence intervals for the overall mean effect size.

Where studies were combined with different scales, we ensured that higher scores for continuous outcomes all have the same meaning for any particular outcome and explained the direction of interpretation.

#### 4.3.7 Summary of findings and assessment of the certainty of the evidence

We did not include Summary of findings or assessments of the certainty of the evidence.

### 4.4 Stakeholders

The following individuals have contributed to the project through the advisory group:  Ronald Amanze; David Truswell—Executive Director of Dementia Alliance for Culture and Ethnicity, Peter Daniels—former Chief Happiness Officer at Humanitas Deventer, Professor Sir Muir Gray—Director of the Optimal Ageing Programme; Iain Lang—University of Exeter; Vicki Goodwin—University of Exeter; Jo Day—University of Exeter; Aideen Young—Centre for Ageing Better; Dylan Kneale—UCL; Ruth Garside—University of Exeter; Claire Goodman—University of Hertfordshire; Tracey Howe—Cochrane Campbell Global Ageing Partnership; Oliver Rashbrook Cooper—Public Health England; Kelvin Yates—AgeUK Cornwall; Nathan Hughes—University of Sheffield; Debbie Hanson—Sheffield City Council; Laura Abbott—Chilypep; Hannah Fairbrother—University of Sheffield; Kerry Albright—UNICEF; Rachel Staniforth—Public Health; Girish Vaidya—Sheffield Children's NHS Foundation Trust; Sally Pearse—Sheffield University.

Members of the ‘Only Connect!’ network also contributed throughout the project. The group has local, national and international members from the care sector, local government, academia, schools and leading organisations involved in providing intergenerational activities. Members of the group brought their experiences of working with older people, people living with dementia and young people with experience of taking part in intergenerational activities.

During the production of the EGM we convened four whole project meetings to include stakeholders and advisory group members to assist with interpretation and understanding, including, making adjustments to the logic model and comments on the report. The second of these meetings identified and confirmed the topic for this review. The fourth meeting incorporated initial feedback on the review findings, logic model and approach to reporting equity characteristics. We have used a newsletter and other methods of sharing ideas and suggestions such as JamBoard to ensure that as many views and perspectives are captured as possible Table 2

**Table 2:** Stakeholder engagement.

Event	Date	Impact
Stakeholder meeting (EGM)	July 2021	Informed/agreed outcomes of interest and dimensions of the map framework
Stakeholder meeting (EGM and review)	Sept 2021	Gave feedback on the map and suggestions for the report.
		Informed/agreed next reviews to take place
Stakeholder newsletter (EGM)	June 2022	Stakeholder asked to think about who to and how to disseminate our work—via Jamboard
Stakeholder meeting (EGM and review)	Sept 2022	Gave feedback on the new review report, informed the equity content and logic model and further plan for dissemination

Abbreviation: EGM, evidence and gap map.

## 5 RESULTS

### 5.1 Description of studies

#### 5.1.1 Results of the search

**Figure 2:**
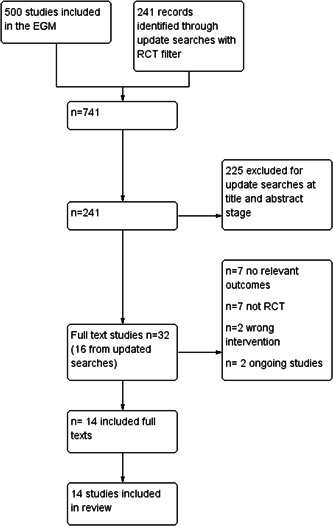
Prisma flow diagram from evidence and gap map (EGM). RCT, randomised control trial.

Using the EGM created in spring 2022 (Campbell Whear, 2023) we found 14 includable RCTs. After two reviewers independently screened these results their data and information was extracted independently using EPPI reviewer EPPI reviewer. All papers that reported a relevant outcome were included in this review. There was one study with two papers (Carlson, 2008; Fried, 2004) that reported that it had collected data on the outcome of depression, but this data was not reported in any of the published papers, we contacted the authors to request this data, but we did not receive a response—therefore this study was excluded as it had no relevant outcome data. The updated searches found 241 references to screen at title and abstract stage of these 16 were then screen at full text stage. After full text screening no additional studies were included though two ongoing studies were identified Digital Buddy: Digital Inclusion for the Elderly; INTEGRITY. Forward and backward citation chasing of these included studies revealed no further includable studies (Figure 2).

#### 5.1.2 Included studies

The number of included studies in this review is 14 (Table 3).

**Table 3:** Study characteristics.

Study	Intervention name	Section A (1) Population characteristics (CYP)	Significant differences at baseline	Section A (2) Population characteristics (OP)	Significant differences at baseline	Who have outcomes been reported for?	Country	Section C—Outcomes (OP)
Carcavilla (2020)	Smile Connect	Age INT M 16.2 years (SD 0.97) CON M 16.3 years (SD 0.48) Recruitment setting secondary school in Italy Total number recruited *n* = 48 (24 per group) Gender Female 100% Ethnicity Italian (otherwise not reported)	None	Age INT Mean 83.8 years (SD 7.89) CON Mean 81.5 years (SD 10.9) Recruitment setting place of residence (care home) Place of residence one of three residential care homes in Spain Total number recruited *n* = 46 (CON 25, INT 21) Gender INT 70% Female CON 52% Female Ethnicity Spanish (otherwise not reported)	None for age, education or cognitive capacity	Children and/or young people Older people	Italy Spain	Self esteem Mental Health
Cardona (2002)	None—Task orientated intergenerational program	Age Mean 16.67 (SD 0.56) Recruitment setting school Total number recruited *n* = 3 Gender Female 66.6% (*n* = 2) Male 33.3% (*n* = 1) Ethnicity not described	Not reported	Age 67–92 range INT Mean 87.57 (SD 3.95) CON Mean 80.83 (SD 8.77) Recruitment setting assisted living facility Place of residence assisted living facility Total number recruited *n* = 13 Gender 4 Male 9 Female Ethnicity not described	Not reported	Children and/or young people intended to be both but only ended up with 3 younger people Older people	USA	Depression Self‐esteem
Chippendale (2015)	Living Legends	Age not reported but in tertiary education 18+ Recruitment setting not reported Total number recruited *n* = 24 students (6 at each site) Gender not described Ethnicity not reported	Not reported	Age CON mean 75.81 years (SD 10.86) INT mean 77.85 years (SD 8.55) Recruitment setting**—**four community based older adult programme sites in New York City Place of residence living in their own apartment Total number recruited *n* = 47 (data only from 39), from four sites 3 were retirement community programs and 1 was a senior centre. Gender CON Male *n* = 1 (5.9%) Female *n* = 15 (93.8%) INT Male *n* = 3 (13%) Female *n* = 20 (87%) Ethnicity White 57.5% African American 36% Middle Eastern 12.5%	None	Older people	USA	Agency MLQ‐Presence Scores
Dawson (2017)	Ageless Play	Age Not reported but range is 6–11 years Recruitment setting school Total number recruited Not reported (matched pairs for the OP so 17?) Gender not described Ethnicity not reported	Not reported	Age Mean age 71 years (SD 8.15), range 55–84 years Recruitment setting Word of mouth, flyers, letter and emails in the surrounding community of the senior centre Total number recruited *n* = 17 (2 removed from analysis) Place of residence Not reported Gender Female 60% Male 40% Ethnicity White (73%), 20% Black/African American (20%) Other (6%)	Significant difference between groups for education only	Older people	USA	
Detmer (2020)	None (Intergenerational music therapy)	Age 3–4years Recruitment setting University based child care setting Total number recruited *n* = 32 (16 in each group) Gender not described Ethnicity not reported	Not reported	Age 72–98 years Recruitment setting Senior living facility (some personal care unit and some memory care unit) Total number recruited *n* = 15 Place of residence senior living facility (some personal care unit and some memory care unit) Gender not described Ethnicity not reported	Not reported	Children and/or young people Older people	USA	
George (2011)	None (Intergenerational Volunteering)	Age 5–6 years and 11–14 years (range 5–14 years) Recruitment setting place of education (The Intergenerational School—TIS) Total number recruited *n* = 32 (two classes of 16) Gender not described Ethnicity not reported	Not reported	Age INT = Mean 85.7 years (SD 5.97) CON = Mean 81.4 years (SD 8.2) Recruitment setting Place of residence—assisted living facility Total number recruited *n* = 15 Place of residence Assisted living facility Gender INT 7 Female 1 Male CON 6 Female 1 male Ethnicity not reported	None	Older people	USA	
Giglio (2006)	None	Age 3–4 years Recruitment setting Pre‐school classroom from the day care centre in the shared site facility Total number recruited *n* = 17 Gender Female *n* = 5 Male *n* = 12 Ethnicity not reported	Not reported	Age 70–97 years Mean 83.10 (SD 5.75) Recruitment setting Place of residence—memory care centre in a nursing home Total number recruited *n* = 29 Place of residence continuous care facility—includes retirement living, assisted living, skilled nursing and memory care services. This population were from the memory care centre which houses those with Alzheimers or dementia Gender Ethnicity not reported	Not reported	Older people	USA	
Gruenewald (2016)	Experience Corps	Age Not reported**—**elementary schools Recruitment setting 6 schools Total number recruited Not reported Gender not described Ethnicity not reported	Not reported	Age 60–89 years Mean 67.4 years (SD 5.9) Recruitment setting Community health fairs, senior centres and housing, life care communities, churches, and community organisations; mailings to members of clubs, AARP, and other retiree organisations, senior housing facilities, and senior centres; and targeted radio stations, including public service announcements and advertising Total number recruited *n* = 702 (INT 352, CON 350) Place of residence Not reported Gender Female 85% Ethnicity 92% Black/African American 5% White/Caucasian 3% other	Not reported	Older people	USA	
Low (2015)	Grandfriends	Age All 4 years old Recruitment setting Preschool class of a childcare centre Total number recruited *n* = 21 Gender Female 48% (*n* = 10) Male 52% (*n* = 11) Ethnicity not reported	Not reported	Age Mean 91 years Recruitment setting place of residence (aged care facility) Total number recruited *n* = 40 Place of residence Three aged care facilities Gender Female 80% (*n* = 32) Male 20% (*n* = 8) Ethnicity not reported	None	Children and/or young people Older people	Australia	
Rook (2003)	Foster Grandparent program	Age Not described Recruitment setting Place of residence (state hospital) Total number recruited Not reported but presumably one for each OP in the intervention group Gender not described Ethnicity not reported	Not reported	Age 60–92 years Mean 70.52 Recruitment setting Community group through mailings and phone calls to older people chosen randomly but based on age and economic stratification Total number recruited *n* = 180 (52 INT 59 CON) Place of residence own home Gender Female 65.6% Ethnicity Caucasian 90% Non Caucasian 10%	Some significant differences across 3 study groups including age, chronic health problems, non‐White participants and SES	Older people	USA	
Sakurai (2018)	REPRINTS	Age Not described but children from six elementary schools, three kindergardens and six public child care centres Recruitment setting school, kindergarten and public child care centres Total number recruited Not reported Gender not described Ethnicity not reported	Not reported	Age Overall Mean 68.0 (SD 4.9) Recruitment setting Recruited from people involved in the REPRINTS study Total number recruited *n* = 177 (only 59 completed all assessments at 6 years) Place of residence Own home Gender Female 85% Ethnicity not reported	Only significant difference in number of years of education	Older people	Japan	
Shkilnyk (1984)	None (type of visiting programme)	Age Mean 12.47 years (Grade 6–8) Recruitment setting school Total number recruited *n* = 72 Gender not described Ethnicity not reported	Not reported	Age Mean 84.57 years Recruitment setting Nursing care home Total number recruited *n* = 54 Place of residence care home Gender not reported Ethnicity not reported	Not reported	Children and/or young people Older people	Canada	
Sipsas‐Herrmann (2000)	SCARE (Student Created Aggression Replacement Education)	Age 11–12 years Recruitment setting School Total number recruited *n* = 194 (172 remained after attrition) Gender Female 102 (92 remained) Male 92 (80 remained) Ethnicity 80% anglo 15% Hispanic 5% other	Not reported	Age INT 60–81 years CON 60–92 years Recruitment setting community and senior centre Total number recruited INT 36 CON 37 (18 assigned to the control group and 18 to the SCARE program plus 37 retired senior citizen volunteers (14 male, 23 female) ages 60 to 92, not actively participating in the project were recruited as non‐trainers for the control condition portion of the cross‐generational investigation) Place of residence own home Gender INT 10 Male 26 Female CON 14 Male 23 Female Ethnicity not reported	Not reported	Children and/or young people Older people	USA	
Thornton (2018)	Senior Change makers	Age Mean 24 years (SD 7.53) college students Recruitment setting University through word of mouth, email lists sent to local university students in health‐related fields of study Total number recruited *n* = 21 Gender Female 81% Male 19% Ethnicity 33% White non‐Hispanic	None	Age Mean 75 (SD 9) years Recruitment setting Place of residence (senior housing residence) Total number recruited *n* = 60 Place of residence Senior housing residence Gender Female 84% Male 16% Ethnicity White non‐Hispanic 70% Hispanic 10% African American 11% American Indian 7% Asian 5%	None	Children and/or young people Older people	USA	

##### Location of studies

Of the 14 the majority (*n* = 10) were conducted in the USA. One study was conducted in each of the following countries: Canada, Japan and Australia with another study conducted across two countries (Italy and Spain).

##### Population characteristics

Although intergenerational interventions by their nature involve at least two population generations, only three of these intervention studies were specifically targeted at both older people and younger people and children. However, outcomes were reported for both generations in half of the studies. The older people involved in the included studies were generally reported as being 65 years and above, although some were targeted at younger ages (50 years and above); others did not describe the age range or indicated a broader characteristic such as ‘retired’ (*n* = 2). There were five interventions that involved young people aged 12–18 years, two that involved young people aged 18–30 years, two that involved children aged 6–11 years, two that involved children aged 0–5 years and two that involved children and young people across more than one age group (one study did not report the ages of the children (Rook, 2003).

##### Study/sample size

The studies sizes ranged from 16 to 702 people with five studies (Gruenewald, 2016; Rook, 2003; Sakuri, 2018; Shkilnyk, 1984; Sipsas‐Herrmann, 2000) including a combined sample larger than 100 people. The number of younger people included in these studies ranged from three to 194 with four studies unable to report the number of younger people included as they were part of a school sample. The number of older people included in these studies ranged from 13 to 702.

##### Equity characteristics

We used the PROGRESS Plus framework (O'Neill, 2014) to guide data extraction of participant characteristics of eligible and targeted populations within the included studies. We hoped to use this information to describe and assess categories of disadvantage based around place of residence, race/ethnicity, occupation, gender, religion, education, socioeconomic status, social capital, other personal characteristics, for example, cognitive decline, and relationship features, however, the information we were able to retrieve was very limited.

From the information we were able to retrieve we could identify that of the 14 studies, four specifically targeted older people with cognitive decline (Detmer, 2020; George, 2011; Giglio, 2006; Low, 2015), one study specifically targeted their intervention for Italian children in secondary school and older people in a residential care home in Spain (Carcavilla, 2020), four studies specifically excluded older people with cognitive decline (Carcavilla, 2020; Cardona, 2002; Gruenewald, 2016; Sakuri, 2018), two studies targeted older people living in their own homes (Chippendale, 2015; Sakuri, 2018), two studies targeted low income areas/populations (Rook, 2003; Thornton, 2017), two studies required a good level of functional language/literacy skills (Gruenewald, 2016; Thornton, 2017) and one study targeted children with a physical disability in hospital (Rook, 2003).

In terms of descriptions of sample populations much of the information we would hope to present in the PROGRESS Plus framework is missing. The most commonly reported characteristics were around race/ethnicity, gender, socioeconomic status, education and other selected personal characteristics like cognitive decline or physical impairments. However, even within these descriptions the information available is limited, and they are not necessarily accounted for in the analysis of the results. We have presented the information we were able to find in Supporting Information: Appendix 2.

##### Intervention characteristics

The majority (*n* = 10) of the interventions were Level 5 (demonstration projects lasting a limited period) (Carcavilla, 2020; Cardona, 2002; Chippendale, 2015; Dawson, 2017; Detmer, 2020; George, 2011; Giglio, 2006; Rook, 2003; Shkilnyk, 1984; Thornton, 2017) of the Depth of Intergenerational Engagement Scale (Kaplan, 2004), with three at Level 6 (Gruenewald, 2016; Sakuri, 2018; Sipsas‐Herrmann, 2000) and one at Level 7 (Low, 2015).

The interventions were largely delivered in‐person with one conducted online (Carcavilla, 2020). Interventions were often delivered in groups (*n* = 6) with some interventions having both group and individual elements (*n* = 6), two interventions were delivered on an individual basis. The interventions were delivered in a range of settings including schools (*n* = 5), care homes (*n* = 4), Hospital (*n* = 1), shared facilities (*n* = 1), nursery setting on a university campus (n = 1), community settings (*n* = 1) and assisted living centres (*n* = 3). Some interventions took place in more than one setting (Carcavilla, 2020; Giglio, 2006; Sakuri, 2018).

The interventions described were delivered over varying timescales ranging from 3 weeks to 1 year, with some studies reporting outcomes over three Rook 2003 and 6 years Sakuri 2018. Most studies (*n* = 11) were conducted over less than 6 months so had only short term outcome data and had no identifiable long term follow‐up plans.

Three interventions took the form of visiting programmes (Rook, 2003; Shkilnyk, 1984), three were school volunteering programmes (George, 2011; Gruenewald, 2016; Sakuri, 2018), two were music‐based interventions (Detmer, 2020; Giglio, 2006), and the rest were task‐oriented involving physical activities in a multigenerational park (Dawson, 2017), reminiscence activities (Chippendale, 2015), activities to reduce aggression (Sipsas‐Herrmann, 2000), learning language skills (Carcavilla, 2020), making local environmental changes (Thornton, 2017) and school project work separate to general volunteering in schools (Cardona, 2002).

The focus of the interventions was also varied, some focused on one particular skill or activity such as developing language Carcavilla 2020, music skills (Detmer, 2020; Giglio, 2006), environmental activities (Thornton, 2017), professional skills/understanding for students (Chippendale, 2015), reading (Sakuri, 2018) and mentoring/anger management (Sipsas‐Herrmann, 2000). Whilst others tended to use multiple activities, such as arts and craft, exercise, sharing meals, storytelling, maths and playing games to encourage interactions more generally.

Those involved in providing/delivering interventions ranged from researchers (Dawson, 2017), volunteers (Sipsas‐Herrmann, 2000), trained students (Thornton, 2017), care home staff (George, 2011; Low, 2015), to occupational therapists (Chippendale, 2015) or music therapists (Detmer, 2020; Giglio, 2006). Some studies did not report who delivered the intervention (Carcavilla, 2020; Cardona, 2002; Gruenewald, 2016; Rook, 2003; Sakuri, 2018; Shkilnyk, 1984).

Six studies reported intentionally tailoring the intervention, these were mainly to be able to adjust to the setting or more functional requirements to allow the intervention to take place (Carcavilla, 2020; Dawson, 2017; Sakuri, 2018; Shkilnyk, 1984; Sipsas‐Herrmann, 2000) and one reported being able to tailor the activity/topic to the interests of the older person (Thornton, 2017).

Other modifications to interventions were largely unclear or not reported. Two studies reported making some changes (Low, 2015; Thornton, 2017) these accounted for changes in the activities or the availability of staff or participants during the intervention.

Intervention fidelity was reported in half of the studies (Chippendale, 2015; Dawson, 2017; Low, 2015; Rook, 2003; Sakuri, 2018; Sipsas‐Herrmann, 2000; Thornton, 2017) but measures of fidelity were focused around participant attendance and attrition. Two studies reported using more detailed measures to inform intervention fidelity (Low, 2015; Thornton, 2017) which included conducting surveys, focus groups and observations informing how the intervention was delivered and received and aspects affecting implementation. However, the impact of fidelity concerns were rarely discussed in the results of the study (Table 4).

**Table 4:** Intervention characteristics.

Study	Section C**—**Outcomes (OP)	Intervention name	Mode of delivery	Setting of the intervention	Frequency of intervention	Duration of intervention	Intervention level	Focus of the intervention	Item
Carcavilla (2020)	Self esteem Mental Health	Smile Connect	On‐line Individual	Care home Schools	Weekly 2 lessons per week each lesson 30 min long	6 weeks	Level 5	Language	
	Intervention logic or underpinning theory Our aim is to provide evidence on the effectiveness of IGPs that involve older adults living in residential care homes as mentors of young adults in an online language‐learning community. Our study promotes intergenerational contact between young adults in secondary schools in Italy and older adults in care homes in Spain. This study's purpose was to examine the effectiveness of a Spanish language educational videoconferencing programme between generations, on the one hand in reducing negative attitudes towards ageing and improving emotional affect among young adults and on the other hand, for improving emotional affect and self‐esteem among older adults. Who provided the intervention Not described What procedures were put in place? The activity was arranged outside the normal activities at the residential care homes and outside curricular classes at the school. Older adult participants were encouraged to help the students to practice and improve their Spanish language skills, allowing them to adopt the role of an expert in the language to be taught. Before starting the programme, they participated in a workshop that prepared them for their role as language teachers, where they learned how to deal with and resolve any problems or conflicts that might arise. Conversation pairs between an older adult/young adult were formed in such a way that everyone had the same amount of contact with the same older adult/young adult, and met at least three different people. This was reflected in the activity calendar that both parties had to guarantee a proper organisation. Older adults in the control group took part in one or more social activities offered to them in their respective settings, in which both they and the older adults in the intervention group were already participating. Some of these activities were board games, bingo, and conversation groups, and they maintained spontaneous contact with young adults visiting relatives in the residential care home. The young adults in both the intervention group and the control group watched 12 videos about culture, traditions, cooking and Spanish geography during their Spanish lessons. Young adults in the control group only practiced Spanish in their formal classes at school, and maintained spontaneous contact with older people. In an initial stage, pre‐intervention data were collected from the participants in both groups for pre‐intervention assessment. This was done by an independent assessor in individual meetings with each older adult participant. The young adults were assessed by answering questionnaires in a self‐administered way on the computer by following the instructions sent to them via email.								
Cardona (2002)	Depression Self esteem	None—Task orientated intergenerational program	Face to face Group	Assisted living centre	Weekly 3 sessions per week for 50–55 min with an extra session on the third week	2–3 weeks	Level 5	Art and craft Exercise Music Other (working together as a team and presenting their work)	
	Intervention logic or underpinning theory Social interactions and influences are often the foundation for the formulation of each individual's identity. Interactions between adolescents and older adults can benefit both groups. The implementation of an after school task oriented intergenerational program may allow positive influences between both groups and may help both groups to increase or improve their sense of self‐efficacy secondary to being able to accomplish a goal and being able to work on a specific and structured task. The purpose of this study is to study the effects of a task oriented intergenerational program on self esteem and depression rates of older adults. Also, to extend quantifiable data on the efficacy of these programs to improve self‐esteem and self‐efficacy of adolescents. What procedures were put in place? On the first meeting an activity to get to know each other was performed, during this activity every participant shared some personal information. On the second meeting only the experimental group was met. A dancing to the Rat Pack and Big Bands (e.g., Benny Goodman) music activity was performed. On the third meeting the experimental group of older adults was divided into three groups. Each group went with a different adolescent. These three groups had to present an activity of their choice to the entire facility. They had to organise the activity and presentation. The activities chosen were: a. exercise group, b. painting group, and c. singing group. For three consecutives meetings they met, organise, practice, etc., to be prepared for the presentations, each group decided how they were going to do the presentations. On the seventh meeting the presentations were performed, and posttest to adolescents were given. Who provided the intervention Not described								
Chippendale (2015)	Agency MLQ‐Presence Scores	Living Legends	Face to face Group	Community setting three naturally occurring retirement communities and one senior centre	Weekly Writing workshop for the first 8 weeks each session 90 min	1–12 months (academic year)	Level 5		
	Intervention logic or underpinning theory Reminiscence and life review have been shown to have a positive effect on the mental health of older adults. Reminiscence involves recalling specific events from the past and can be done silently or through the spoken word. Life review, which is more formal than reminiscence, involves a systematic review of life events from childhood to the present and includes an integrative component in which people reflect on their lives as a whole. Evidence suggests that life review has a larger effect than reminiscence in improving depressive symptoms. Moreover, life review through writing has been found to be more effective than oral life review. We hypothesised that older adults who participated in Living Legends would have an enhanced sense of purpose and meaning in life compared with older adults who participated in life review writing alone. Evidence that volunteering enhances mental wellbeing and life satisfaction. What materials were used? writing workshop to write about life chronologically and receive feedback on writing technique. Integrative component**—**in which participants write about how their life experiences have shaped who they are. During the intergenerational intervention**—**each older adult participant read one piece of his or her work self‐selected from the preceeding 8 week workshop. After each reading a guided discussion took place between the older adults and students about the content of the writing. What procedures were put in place? all took part in the 8 week ‘Share your Life Story’ life review writing workshop. The health science students were given a brief orientation that included program expectations and tips for communicating with people who have hearing loss. Who provided the intervention Writing work shop led by an experienced occupational therapist (PI on project).								
Dawson (2017)	Quality of Life Self‐efficacy Self worth Enjoyment Personal growth	Ageless Play	Face to face Individual	Shared facility/other multigenerational play park outside the senior facility	Weekly 1 h sessions	5 weeks	Level 5		
	Intervention logic or underpinning theory Intergenerational programming between children and older adults have been shown to make significant contributions to older adults’ overall wellbeing. This study focuses on comparing a control group, an active control group with those participating in an on‐going exercise class offered at a senior centre, and an experimental group taking part in an active intergenerational program on a multi‐generational play park. It is projected that users of multi‐generational play parks integrated with stealth exercise will experience a boost in energy and reap benefits of fresh air and nature while undergoing simple recreational and leisure activities. An active intergenerational program designed for the use of multi‐generational play parks can contribute a greater positive impact for older adults to adopt the concept of active aging and maintain healthy lifestyles. Significant benefits provided by intergenerational activities for older adults are (1) the experiences that come with it can be ideal for older adults to prevent and resolve issues that occur in late life, and (2) intergenerational activities that are designed to help youth successfully assist older adults in accomplishing certain life stages outlined by Erikson, such as integrity versus despair. Evidence also shows that playing with children allows older adults an opportunity to reminisce about their past childhood, while children receive an enriched learning experience from interacting with positive role models. In this research it is also stated that, ‘Play, a basic activity of childhood, when combined with older adults in an intergenerational setting, opens a new gateway to intergenerational programming’. Because of this, intergenerational programming at a multi‐ generational play park is highly likely to foster interaction, teamwork, and relationship building between older adults and children. Focuses on active aging and exercise guidelines to improve physical and mental health. What materials were used? This intergenerational program that will take place on the multi‐generational play park outside of the senior centre is designed to foster intergenerational collaboration and build relationships between older adults and children. The program comprises not only of this teamwork establishment, young and older generations working together to accomplish set goals for planned activities, but also ways for older adults to stay active in an innovative way. This 5‐week long program focused on different themes each week such as: introductions and teamwork, continued teamwork, strength, balance and be creative, which utilises all components of the multi‐ generational play park. What procedures were put in place? Those who were randomly selected to participate in Ageless Play also had to attend an Ageless Play orientation, which lasted no more than 45 min. The general layout of each 1‐h activity session consists of warm up laps, warm up stretches, the main activity, free play, and a cool down all of which the child and older adult pair executes together. Who provided the intervention Run by the researcher								
Detmer (2020)	Self esteem Intergenerational interactions	None (Intergenerational music therapy)	Face to face Group	Childcare facility on University site	Weekly 2 sessions per week each 30 min long	12 weeks	Level 5		
	Intervention logic or underpinning theory It is well established that intergenerational programs improve cross‐age attitudes and meaningful interaction between the two groups; however, there are many unanswered questions as to how they can affect academic skills, physical functioning, self‐worth, and social interactions in one or both age groups. Therefore, the purpose of this study was to identify the effects of an intergenerational music therapy program on children's literacy, older adults’ physical functioning and self‐worth, and interactions between the two age groups. What materials were used? Detmer 2020. pdf: Page 7: ‘Materials for this study included video/photography equipment, musical instruments, craft items, visuals/props, child and adult chairs, accelerometers, and storybooks. Four GoPro® cameras were mounted in the corners of the room: two at frogs‐view and two at birds‐view. These allowed recording of every session from multiple angles to be used for behavioral observation and interaction analyses. Instruments included paddle drums, egg shakers, boom‐ whackers, ocean drums, scarves, rhythm sticks, and two guitars for the music therapists’. ‘All older adult participants wore a Fitbit® on their wrist for the duration of the 12‐week study period to track physical activity. Other mate‐ rials included toy echo microphones, a large parachute, floor tape for a walking path, laminated visuals of the alphabet, and pictures to correspond with some of the movement and storybook activities. Twelve storybooks were also used in the study. To determine which books to use, the authors**—**with help from fellow music therapists, teachers, early childhood blogs, and social media threads—’ ‘created a list of children's books that were either about music, set to music, or commonly used by music therapists/teachers for a total of 293 books. This list was then reviewed specifically for age ranges to ensure they were appropriate for 3‐year‐old children, after which 144 books were discarded. Then, the remaining list of 149 books was given to the classroom teachers of the child participants and the teachers were instructed to cross off any book used or available in their classroom, leaving 82 books'. What procedures were put in place? Each session followed Gooding's (2013) evidence‐based early‐childhood music therapy group session format. The researchers designed 12 unique session plans corresponding to a theme based on each of the books. Each session plan was facilitated one time over the first half of the study period and then repeated during the second 6 weeks. A research assistant rode the bus with the older adults to the pre‐ school for each session. During the drive, the research assistant handed that day's storybook to each adult and instructed them to read along to prepare them for the upcoming activity and interaction to come. The older adults arrived 10 min early each day to allow time to find their chair and situate their walkers/canes outside of the group circle. During this time, the co‐therapist facilitated the gathering song, ‘It's Time for Music’, to orient the adults and allow for material distribution. Next, the therapists began the greeting song (3 min), ‘How Do We Say Hello’, to promote intergenerational interaction. The song included embedded prompts allowing the participants opportunities to suggest different ways to greet one of their grandfriends (e.g., wave, high‐five, or give a fist bump). A transition song, ‘We're all Done with (Singing)’, was then used to cue the participants for the next activity, which was movement based (e.g. ‘Head, Shoulders, Knees, and Toes’) (5 min) to improve the physical functioning of the older adults. This often required rearranging of the chairs to create an open space. In an effort to offer structure and a visual boundary for participants, orange tape was used on the floor to create a movement path around the perimeter of the room. The transition song was then sung again while chairs were moved back to their original position. Instrument play/music making (5 min) to promote intergenerational interaction followed by storybook singing (5 min) to improve literacy skills was next. During the singing of the text, the participants followed along in the book, which was held by the therapists and assistants positioned around the room. Storybook reading (5 min) to improve literacy skills followed. For this activity, each adult received an individual copy of the storybook and was instructed to read the book to their children. After the storybook reading, the lead therapist led a chant‐based activity, ‘We're Going on a Letter Hunt’, in which three to four different alphabet letter visuals were held up one at a time, cueing the children to find the letter in their book. All 26 letters were used in this activity at least twice over the course of the study period. The therapists and assistants moved around the room during this activity to reinforce and assist the children, if necessary. After the children successfully pointed to the indicated letter, they were asked a follow up question, ‘What sound does the letter ___ make?’ After the letter hunt, the lead therapist again used the transition song while collecting the books, signalling the end of the session. To close, the goodbye song, ‘Let's All Say Goodbye’, was sung to promote intergenerational interaction. All participants were encouraged to use their body (e.g., wave, shake hands, or give a hug) to say goodbye to one another. After the song, the children were instructed to quietly line up and follow a staff member back to their classroom. All walkers/canes were given back to the adults and they also left the room and walked to their bus outside (Detmer & Kern, 2017). Who provided the intervention Each session was co‐led by two board‐certified music therapists and took place at the childcare facility in a large open room. Staff and student volunteers also helped.								
George (2011)	Depression Anxiety Quality of Life Agency Self‐efficacy Cognitive activity	None (Intergenerational Volunteering)	Face to face Group	Schools (The Intergenerational School)	Weekly In alternating weeks, partici‐pants served as mentors during hour‐long visits with a kindergarten classroom and a 6th grade classroom	5 months (20 h volunteering per volunteer)	Level 5		
	Intervention logic or underpinning theory A subset of research has established that older adults who form relationships with children through intergenerational volunteering programs seem to experience specific benefits, such as improvements in health status and well‐being, increased activity, strength, and cognitive ability, the creation of meaningful relationships, enhanced self‐ esteem, increased social capital, and better psychological functioning. The Intergenerational School is structured around the ideology that people of all ages can learn alongside each other throughout their life spans. This commitment extends to older persons in the long‐term care community—some with memory loss—who are invited to serve as ‘mentors’ with the students. The school is the first known educational institution in the world to create a formal mentorship role for persons with dementia. What materials were used? Not described What procedures were put in place? Previous to the intervention, the researcher convened separate pre‐intervention meetings with all participating elders and children, and with the teachers of the two host class‐ rooms, to explain the study design and field questions from all participants. This provided participants and staff with an opportunity to explore feelings and apprehensions about the pending interactions, identify the existence of common stereotypes, and ascertain factual information about the study. All participants in the intervention group were involved in direct volunteering experiences with children aged 5–14 years. In alternating weeks, participants served as mentors during hour‐long visits with a kindergarten classroom in which they interacted with children and engaged in singing and small‐ group reading and writing activities, and a 6th grade classroom where they broke into smaller groups with 2–3 students and participated in intergenerational life‐history reminiscence sessions. The control group met eight times at JP for a peer education seminar called ‘Successful Aging: Reclaiming Elder‐ hood’ for a total of approximately 12 h. Workshops facilitated by JP staff focused on the following themes: learning, wellness, love, creativity, spirituality, life options, ethics and beauty, and life quality. Control group participants were given eight home‐work assignments between each session that were intended to take 1 h each to complete; ultimately, the output of volunteer hours for the JP group was equal to the intervention group at TIS. Who provided the intervention The study was undertaken in partnership with The Inter‐generational School (TIS), an organisation that fosters intergenerational interaction between its 200 students and older adults in the Northeast Ohio community, and Judson Park (JP), an assisted living facility in Cleveland that is registered in the Eden Alternative. Classroom teachers with help from school volunteer coordinator and director from the assisted living facility.								
Giglio (2006)	Behaviour	None	Face to face Group and Individual	Assisted living centre Memory care unit was secure	Weekly 30 min per session one morning per week	8 weeks	Level 5		
	Intervention logic or underpinning theory The following study was designed to examine the effect of a music therapy intergenerational program on cued and spontaneous behaviours of older adults with dementia. Research in music therapy in the geriatric field has shown that many different music therapy activities have been beneficial with working with older adults with dementia. The most common areas of investigation have included singing, creative movement and instrumental rhythmic playing. Reminiscing has also been received positively for its use with older adults. Singing evokes the use of memory with recalling the words or familiar melody of a song, and may trigger remembering where, when and/or who sung the song to them. Automatic language and memory skills are also challenged during complete the song phrase/title before singing a preferred and familiar song. When music is introduced into exercise, it helps to invigorate and encourage a person due to the music's rhythmic qualities. It is this strong rhythm in music that helps to organise and structure the movements instructed, as well as make it easier to predict when a person will be expected to move or transition into a new movement. For persons with dementia, this type of intervention has been shown to have the highest response and purposeful participation levels. Groene II et al. suggested that this might be due to the constant visual prompting that the older person is receiving, making it less cognitively challenging to complete the task. Several studies have revealed that instrument playing ranked slightly higher than exercise with regard to purposeful activities with high participation levels for persons with dementia. Research has also revealed that older persons with dementia are able to feel or tap out a rhythm, especially if the beat is strong and is utilising a vibrotactile instrument. When using music to reminisce, the music therapist is using client‐preferred songs to help conjure memories to share stories. What materials were used? Not reported—Each 30‐min session was theme‐based to promote reality orientation, to encourage reminiscing and to encourage physical and/or verbal interaction with others. What procedures were put in place? Once permission had been obtained by the Executive Director, the Child Development Center Director, and the Lifestyles Director of the Foxwood Springs health care centre, the guardians or POAs of the children and older adult participants were contacted and offered the opportunity to participate in the study. If interested, the guardian or POA of the participant signed a statement of informed consent and consent for videotaping. Each group met for 30‐min one morning a week for 8‐weeks. Classroom teachers invited a maximum of 10 out of the 17 students to the intergenerational music therapy group each week so that interactions with older adults could happen on a one‐to‐one basis. Individual staff in‐service meetings were scheduled for the special care unit, child development and lifestyles staff to inform them about the study 1 week before the start of the sessions. Opening Application (3 min) Older adults will be seated in a semi‐circle. Children will walk in a line around the circle and shake each older adults hand and then proceed to sit in the middle of the semi‐circle when finished as the music therapist sings the hello song. ng (5 min) The music therapist will provide a question for the children to ask the older adults. Music therapist sings a song to incorporate answers to the question. Music therapist will ask several pairs of the older adults and children to report their answer. Physical Exercise (10 min) A. Movement (5 min.): Older adults will be paired with a child. Exercises will be done with or without props to recorded music. B. Instrument Playing (IP) (5 min.): Children and older adults play instruments individually to recorded music. Singing (5 min) Children sing to older adults. Older adults sing to children. Children and older adults sing together. All songs will incorporate the theme of the day. Closing Application (2 min) Children will face his/her older adult and sing a good‐bye song to him/her. Who provided the intervention A board certified music therapist. During group sessions, staff members were asked to assist participants with such things as toileting, wandering, physical guidance of 1‐2 older adult that were assigned to that staff member, as well as pairing children and older adults together, if needed.								
Gruenewald (2016)	Agency	Experience Corps	Face to face Group and Individual	Elementary school	Daily 15 h per week	Volunteers had to commit for 1 school year but were encouraged to stay for 2	Level 6		
	Intervention logic or underpinning theory Not reported (see Fried et al., 2013) The BECT is a dual effectiveness trial of the impact of the EC program on older adult participants and on children in public elementary schools receiving the program. EC is designed to attract older adult participants through the opportunity for generative engagement and then to operate via cognitive, physical, and psychosocial pathways to enhance the health and well‐being of older adult volunteers while simultaneously promoting the academic and psycho‐ social well‐being of elementary schoolchildren and the climate and social capital of the school and community in which the EC program resides. We theorised that a program could be designed to provide older adults with generative roles that improve academic success of young children, and that this would be attractive to diverse older adults who would stay in such roles long‐term if the impact was high and roles were meaningful. Further, we theorised that if evidence‐based health promotion was embedded in the program, targeting multiple behaviours to create additive or synergistic benefits, communities could be provided with long‐term, ‘high dose’ health promotion and prevention benefits, reaching older adults not reached by traditional health promotion programs. Experience Corps (EC) is a civic engagement program designed to harness the time, energy, and wisdom of older adults to improve academic outcomes of elementary school children. EC volunteers serve in a variety of roles designed to meet important unmet needs of a school as determined by the school principal, commonly assistance with literacy and math instruction and providing children with attention and guidance needed to support positive behavioural development. EC is designed to be an intergenerational win‐win enhancing the academic and sociobehavioral well‐being of elementary school children and providing older adults with an opportunity to fulfill generative desires of meaningfully contributing to others and promoting the next generation while simultaneously exposing older volunteers to social, cognitive, and physical activity associated with more favourable trajectories of health and functioning in later life. More positive self‐perceptions of generativity are correlated with lower levels of negative affect and depressed and anxious mood in middle‐aged and older adult samples. Greater self‐perceptions of generativity are also linked to more positive psychological well‐being in both mixed‐aged and older adult samples. What materials were used? Not reported What procedures were put in place? Those randomised to EC are assigned to serve for at least 1 year in a public elementary school, with grades Kindergarten through the third grade. Who provided the intervention Not reported								
Low (2015)	Quality of Life Behaviour/engagement Community Agitation Sadness Pleasure	Grandfriends	Face to face Group and Individual	Assisted living centre	Weekly once per week in 45 min sessions	12 weeks	Level 5 (Level 7? As co‐located site?)		
	Intervention logic or underpinning theory The Grandfriends program was developed collaboratively between preschool staff, nursing‐home recreational staff, and the research team. Grandfriends was designed to be enjoyable, encourage interaction, and develop relationships between the generations by encouraging both groups to work together towards a common goal. The program also had to meet the programming needs of the aged‐care facility and address outcomes in the Australian early childhood framework. The aim of this study was to evaluate outcomes of Grandfriends, an intergenerational program for people living in nursing homes as a result of their dementia symptoms and children attending a preschool colocated within the facility precinct. We hypothesised that older adults with dementia‐causing conditions will be more engaged during the intergenerational program than during an activity provided as part of usual care at which the children are not present. The rationale was that increased engagement during the activity would meet needs for meaningful activity and social engagement and result in improvements in quality of life and sense of community and in decreased agitation among those with dementia symptoms. What materials were used? Not described What procedures were put in place? The program involves pairing each child with a ‘grandfriend’ and participating in a range of activities together such as discussions (e.g., similarities and differences), craft (e.g., collage), and games (e.g., bingo). Who provided the intervention Educators from the day care centre and nursing‐home staff jointly facilitated the activities.								
Rook (2003)	Depression Self esteem Loneliness Community	Foster grandparent program	Face to face Individual	Hospital	Daily 4 h 5 days per week	3 years	Level 5		
	Intervention logic or underpinning theory The current study investigated the effects of involvement in a social role that was conceptualised as contributing to older adults’ psychological well‐being through two different pathways: first, by creating conditions hypothesised to be conducive to friendship formation, the participants’ activities were expected to facilitate the formation of new social ties and thereby enhance their psychological health; and second, by providing a context in which participants regularly helped to nurture and care for a developmentally‐disabled child, the program was expected to bolster feelings of self‐worth. We anticipated that such regular contact, organised around shared activities, would facilitate the acquaintanceship process. Moreover, the program involved frequent contact extended over a sufficiently long period of time to allow such relationships to emerge gradually and in the relatively natural and familiar context of shared activities. For these reasons, we expected involvement in this program to contribute to the formation of new friendships among participants and, in turn, to greater emotional well‐being. What materials were used? Not described What procedures were put in place? Those assigned to the FGP condition were assigned a primary ‘client’ (a developmentally‐disabled child in residence at the state hospital) and, after receiving a standard orientation and training, were given duties typical of a foster grandparent. The foster grandparents worked 4 h per day for five mornings each week, and received a modest stipend for their work (set by federal policy to correspond roughly to minimum wage). In addition, the foster grandparents ate lunch (provided by the program) together at the end of each workday. Those older adults assigned to the AGP group continued their participation in the meals and activity programming at the regional nutrition centres. They received a monthly stipend of $50, to both provide a symbolic control for the effects of the monetary compensation received by the foster grandparents, and to compensate them for the time spent in the annual assessments. They understood that they were important participants in the research study but that they were not on a waiting list for eventual inclusion in the Foster Grandparent Program. Participants in the CS group received $50 annually, plus travel expenses, to compensate them for the time and costs associated with the annual assessments. Who provided the intervention Not described (older adults)								
Sakurai (2018)	Cognitive activity	REPRINTS	Face to face Group	Schools Other (kindergarten and after school childcare centre)	Weekly visited once every 1–2 weeks for 15 min/30 min to 2 h	6 years	Level 6		
	Intervention logic or underpinning theory an intergenerational program that involves engaging older adults in reading picture‐books to kindergarten and elementary school students, with the expectation that it will help maintain or improve the cognitive and physical functions of older adults. The program is expected to establish new social networks with members, children, teachers, and program staff, and contribute to the healthy upbringing of children. The REPRINTS program was designed to bolster intellectual ability and social function by exercising language, mental flexibility, and working memory and increasing social interaction via reading picture book activities to children. Because participants in social engagement programs may be relatively healthy, long‐term observation is needed to correctly estimate the program's benefits. Here, using a 6‐year follow‐up, we determined whether the REPRINTS program, which is a productive social engagement intervention, can prevent age‐related hippocampal atrophy and cognitive decline compared with control participants. What materials were used? Not described—further info in other papers What procedures were put in place? REPRINTS participants trained to read picture books 3.3 times per week, on average. Conducted group activities (6–10 members per group) in 6 elementary schools, 3 kindergartens, and 6 public childcare centres. At kindergartens, partic‐ ipants played hand games (e.g., exercising the hands to a rhythm or song) and read 3 or 4 picture books for 30 min per class. In elementary schools, they read 1 to 2 picture books in the morning for 15 min per class. In addition, they sometimes (approximately once every 1–2 weeks) read picture books for children during lunch breaks. At public childcare centres, they freely read picture books and played with children after school. Each group had regular meetings before and after reading sessions to share information, discuss ways to improve the quality of reading techniques, and train in reading picture books Who provided the intervention Older people (not otherwise described)								
Shkilnyk (1984)	Life satisfaction Intergenerational interactions Social activity	None (type of visiting programme)	Face to face Group and individual	Care home	Weekly 1 h per week	20 weeks	Level 5		
	Intervention logic or underpinning theory Social intervention programs have a positive effect on elders, many variables connected with life satisfaction, intervention programmes can result in attitude change in adolescents. What materials were used? Not described What procedures were put in place? Parents gave consent for their children to volunteer. Careful matching of adolescent and elder. three groups: Info ex group had an information and orientation package and then visited elders, Info group only had the information and orientation package, control group received nothing the children were ‘matched to elders’—matching involved developing a character synopsis for each of the elders and the children, then the programme director of the care home and the counsellor from the school and the investigator agreed on who was matched with who. once matched the elder and younger person were each given the information and orientation package before meeting each other. there were two lectures on what it feels like to get old and how to get along with the elderly, and a tour of the facilities. They were then introduced to their elder person the time visiting could be spent playing games, having tea, doing craft or just chatting. Every 3 weeks the investigator held small group meetings for the young people to share any learning or concerns Who provided the intervention Not reported								
Sipsas‐Herrmann (2000)	Agency Self‐efficacy	SCARE (Student Created Aggression Replacement Education)	Face to face Group	Schools	Weekly twice per week for 45 min each session	8 weeks	Level 6		
	Intervention logic or underpinning theory The SCARE program is a treatment package that combines a variety of effective treatment protocols into one program that has demonstrated it's effectiveness. According to Herrmann and McWhirter, the treatment package is based on the tenet that angry and aggressive individuals hold biased, hostile attributions or beliefs about the intentions of others. The SCARE program perspective is that teaching reattribution of perceived offenses and the control of resulting anger is key to preventing violent and aggressive acts from occurring. This present study sought to further validate the SCARE program using the senior citizen population as trainers as opposed to graduate students that were used in the initial validation study. The goal of this study was to capitalise on this very useful and abundant resource and at the same time assess the versatility of the SCARE program by examining if it can be successfully delivered to youth by an average citizen. Its objectives include (a) teaching young people about emotions, including aggression and anger, (b) helping young people recognise alternatives to violent behaviour and aggressive responses, and (c) encouraging youth to make good decisions in response to provocative situations What materials were used? The Enter Here program was utilised as the control condition in this study. This program is a 16‐session video‐based vocational exploration program designed to help students at the beginning stages of formulating their interests and ideas regarding future vocational work. The program has been shown to be effective in moving students towards greater career maturity and career self‐efficacy. Each session consisted of two videotaped presentations on particular jobs, followed by facilitator‐led discussions of each video. The length of each video presentation was approximately 7 min each, and the subsequent discussion entailed the remainder of the class period. The SCARE Program Session # 1: Recognizing Anger and Violence Session #2: Family/Friend Tree Managing and Reducing Anger in the Self Session # 3: Internal Responses to Anger Session # 4: Anger Journal Session # 5: Reducing Arousal Through Positive Self‐Statements Session # 6: Systematic Deep Breathing Session #7: Progressive Relaxation Session # 8: Exercise Defusing Anger and Violence in Others Session # 9: Session # 10: Session #11: Session # 12: Session #13: Session # 14: Session #15: Session #16: Creative Alternatives to Violence Paraverbal Techniques ‘1’ Instead of ‘You’ Reflections Proxemics (Personal Space) Kinesics (Body Language) Appreciating Diversity (The Hand Clasp Exercise) No Violence Contracting What procedures were put in place? Students received the intervention during their assigned P.E. class twice a week for 8 weeks. Sixth‐graders within each participating P.E. class period were randomly assigned into groups of 8–12 students that either received the experimental condition (SCARE program) or the control condition (Enter Here program). There were nine groups in each condition. The middle school was responsible for notifying the parents of their child's participation in the study. Pretest measures were collected from the students during their regularly scheduled intervention times before the beginning of the intervention. Although the measures are self‐report instruments, each item was read out loud to students and questions were permitted when pertaining to comprehension of the item. Follow‐up measures were collected from students 8 weeks after the post test to assess for maintenance of anticipated treatment effects. Two facilitators were assigned to each group of students, and both were expected to attend every session when possible. During illness or emergencies, those trainers that lost their partners mid‐intervention were able to call a trainer from a different time period to act as a substitute for that day. Following the 8‐week intervention, trainers were asked to convene for a short debriefing session as well as completion of the MPD and narrative for post test purposes. To ensure the integrity of both the experimental and control treatments, training sessions were held to ensure that all trainers were equally proficient in administration procedure and ability. Training sessions consisted of brief overviews for each of the 16 SCARE sessions, mock session administrations, and similar control treatment instruction. Additionally, written training protocols for both the experimental and control treatments were furnished to facilitators. Half‐way through the intervention (4 weeks), a third ‘booster’ training session was held to review the remaining material as well as answer any questions the trainers had. This investigator was present on the school campus for the first week of the intervention to ensure all groups were running as intended. Additionally, throughout the 8 ‐week intervention, periodic random spot checks of the groups were held. Who provided the intervention Older adults as trained volunteer trainers and volunteer facilitators.								
Thornton (2018)	Self‐efficacy	Senior Change makers	Face to face Group	Care home	Weekly 1 h session per week	8 weeks	Level 5		
	Intervention logic or underpinning theory Intergenerational community building programs may provide an ideal mechanism to teach older and younger adult community members to advocate for improvements to their physical activity environments. Intergenerational community building projects aim to engage young people and older adults in projects that benefit the community and concurrently empower participants. The joint advocacy efforts of younger and older adults can demonstrate the possibilities of civic engagement as a grass roots movement, and show an inclusive approach to community building that crosses boundaries of age and income. The primary aim of the study was to assess the efficacy of an advocacy program in comparison to a physical activity program to increase seniors’ advocacy skills and confidence. We hypothesised that the using ecological framework an advocacy program would produce greater improvements in seniors’ advocacy skills and confidence at 8 weeks as compared to the physical activity program**—**Intergenerational groups may be effective advocates since both older and younger adults are major stakeholders in their communities’ physical activity environments, and both groups can contribute unique skills and experiences to the advocacy process. What materials were used? Not described What procedures were put in place? All student participants underwent a training before the commencement of the 8‐week program. Separate half‐day trainings were held for students assigned to the advocacy sites and students assigned to the physical activity sites. The separate trainings were necessary to avoid contamination across conditions. To create a ‘high contact’ intergenerational experience, students in the advocacy condition were trained to participate in all activities with the seniors and interact with the seniors as much as possible. Students in the physical activity condition were trained to serve as assistants to the researchers. Both trainings included sensitivity training to address issues specific to working with older adults. The participants in the advocacy intervention underwent an 8‐week advocacy program. The researchers prepared advocacy curriculum materials in advance, but the focus of the curriculum was tailored to the interests and types of projects selected by the participants. Both advocacy groups completed an environmental assessment using the MAPS‐Mini tool during Week 3 of the advocacy program. Participants at each advocacy site were divided into four small groups (2–3 seniors and 2 students) and each group was assigned an audit route in the neighbourhood. The following week the advocacy groups discussed the audit results and each group brainstormed 11–12 potential advocacy issues related to improving the pedestrian environment. From that list of issues, the groups each selected four to five priority issues that they wanted to present to the traffic engineer during week five of the program. The traffic engineers, who worked for San Diego's Department of Transportation, were asked to provide feedback regarding the feasibility of the projects and recommendations on how to advocate for change. The participants selected one or two group members to present each issue to the traffic engineer. The traffic engineers recommended that the seniors submit online requests through the City's ‘Get it Done’ website. They provided the senior participants with departments and phone numbers to call for future advocacy issues. The engineers provided information regarding the process used by the City to evaluate, prioritise, and fix problems reported by citizens. After presenting issues to the traffic engineers, the older adult participants worked with students to make online requests regarding pedestrian advocacy issues of their choosing. The students helped the seniors type the description and upload photographs. The final 2 weeks of the program were spent learning additional advocacy skills, creating action plans, and thinking about how to handle common advocacy challenges. Participants reported on the advocacy actions they had taken and received feedback from the group. To create a ‘high contact’ condition pursuant to Contact Theory (Table 2), the students in the advocacy condition were encouraged to work with the older adults on advocacy projects. Activities were designed to create equal group status, common goals, and intergroup cooperation. During the field audit, students assisted the older adults by helping them complete the MAPS‐Mini audit, and taking pictures of physical activity barriers. The pictures were sent to the City Department of Transportation as part of the online requests to fix selected pedestrian‐related issues. The students and older adults worked together to draft and submit online requests. During in‐person meetings, the research team gathered information relating to eligibility criteria. We assessed willingness to participate, addressed questions and concerns, discussed recruitment potential, and evaluated site facilities. To meet requirements, students receiving internship credit assisted researchers with data input and cleaning. They also completed a dissemination project to share their internship experience through video, writing, or art. The student dissemination projects were shared with the senior participants during the final week of the intervention. Participants were informed that they would receive a $20 gift card for participating. The researchers then worked with the site to determine the day and time of the weekly group meetings. At each site, meetings were held at the same day, time, and place each week. The Coordinator identified challenges including working with non‐English speaking participants and failing to set clear expectations regarding program duration and incentives. The Coordinator also identified factors that facilitated successful advocacy, such as engaging on‐site staff, developing group identity, and providing food at each meeting. The research team conducted informal interviews with two social workers who worked at low‐income senior housing sites. The social workers provided valuable information on everyday issues that low‐income older adult participants might face. Who provided the intervention Trained students—Group sessions were taught by four members of the research team, which included two public health doctoral students, an individual with master's degree in City Planning, and a resident in preventive medicine.								

#### 5.1.3 Excluded studies

We obtained our included studies from the EGM which has an RCT filter and filters for relevant outcomes, so no studies were excluded through screening, however, two studies were excluded from analysis as they provided no data on an outcome they reported (Carlson, 2008; Fried, 2004). From the update searches 16 studies were excluded at the full text stage because they did not report a relevant outcome (*n* = 7), they were not RCTs (*n* = 7), they were not an includable intervention (*n* = 2) or were ongoing studies with no data yet available (*n* = 2).

### 5.2 Risk of bias in included studies

**Figure 3:**
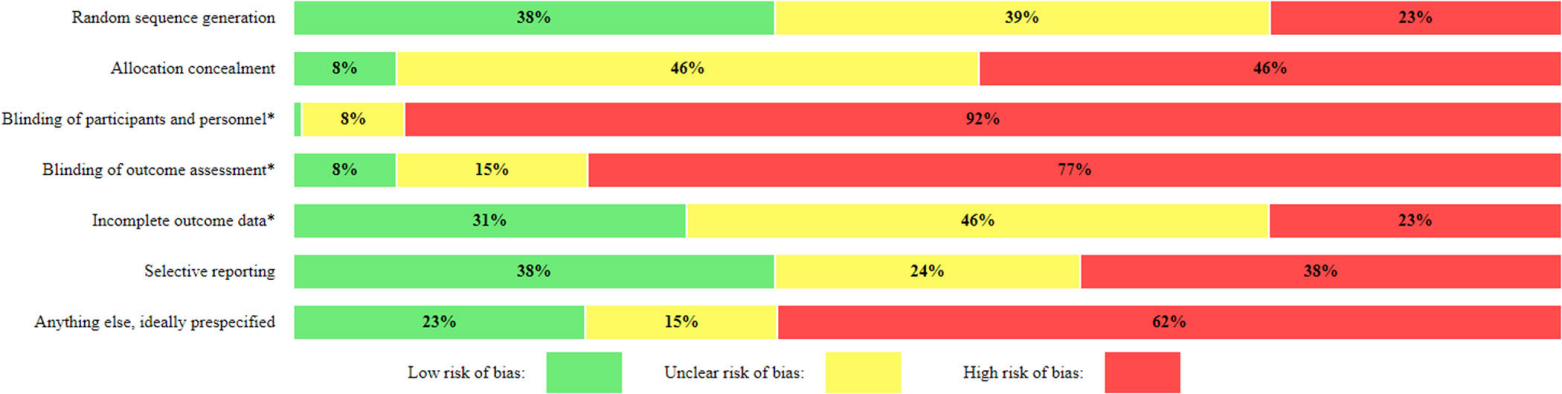
Risk of bias summary.

We used the Cochrane Risk of Bias tool to understand the level of potential bias in these RCTs (Figure 3).

**Figure 4:**
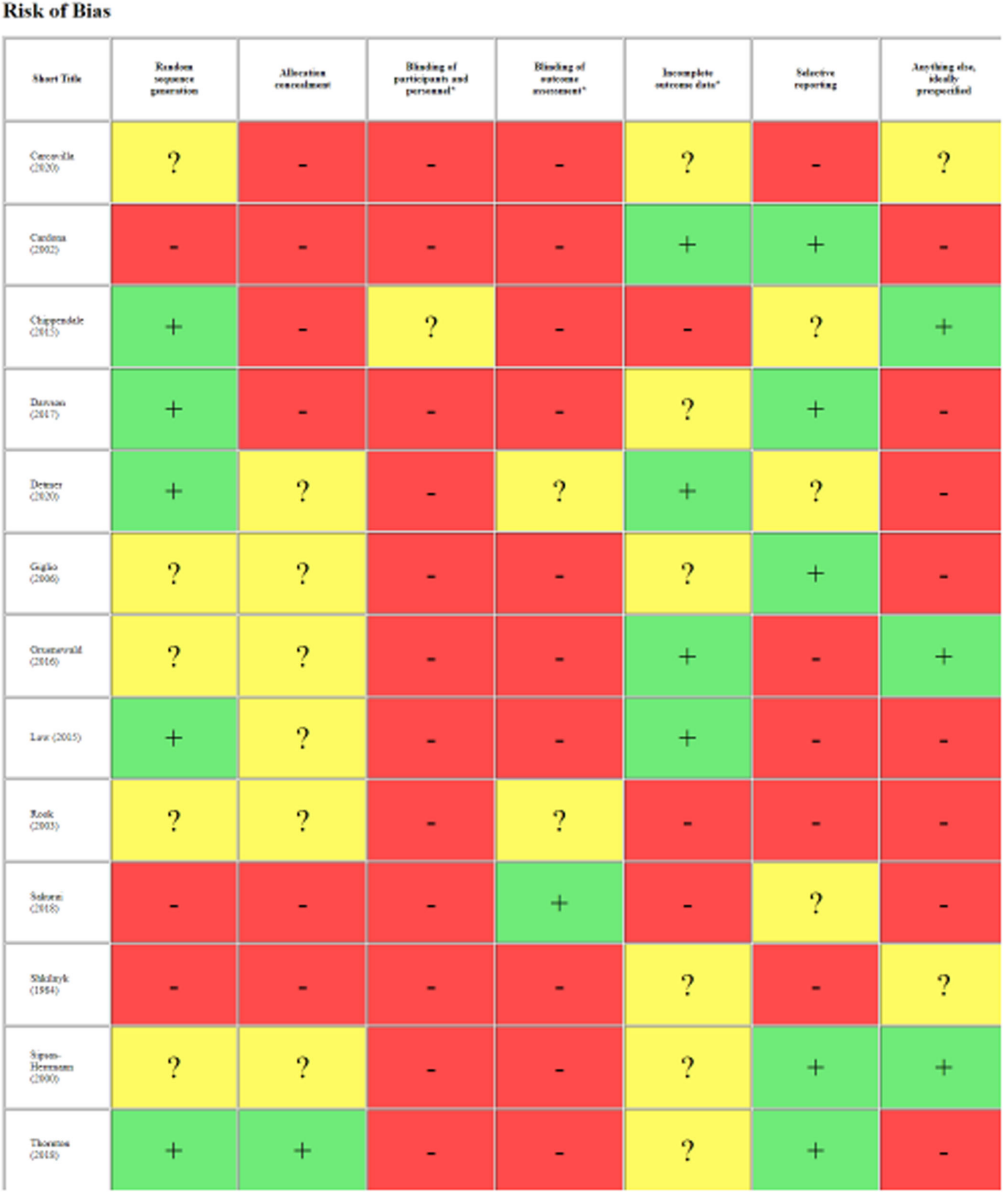
Risk of bias table.

All the included studies are at high risk of bias. Most studies scored positively (with low risk of bias) on only two items or fewer (out of seven items). Areas of particular concern are blinding of participants, personnel and outcome assessment, allocation concealment and sample size. Blinding of participants and personnel is particularly difficult in socially based complex interventions where it will be obvious to participants and those around them that they are in an intervention (of some kind). This risk can be appeased to some extent by blinding outcome assessors but in most of the included studies the tools used to gather data were in self report form or the person collecting the data was aware of the participant's grouping. Even if this domain is excluded from the risk of bias analysis the overall judgement for the risk of bias in these studies would not change substantially. The reporting of methods of allocation concealment was absent in most studies, studies were generally small (from 16 to over 700) and sample size calculations were reported in only two of the 14 studies (Chippendale, 2015; Low, 2015) (Figure 4).

### 5.3 Effects of interventions

**Note**: ‘Effects of interventions’ heading will be removed at publication stage

#### 5.3.1 Synthesis of results

We have structured this section of the report based on the original research questions asked.

*Research Question 1: What is the effect of intergenerational interventions on the wellbeing and mental health of older people?*

##### Primary outcomes

The range of outcomes reported in the studies included in this review varied greatly. The following six outcomes: depression (*n* = 3), anxiety (*n* = 1), quality of life (*n* = 2), self‐esteem (*n* = 4), agitation (*n* = 1) and loneliness (*n* = 1), reflect the primary outcomes that we aimed to capture to assess mental health and wellbeing in older people. Social isolation was not captured in the included studies.

**Figure 5:**
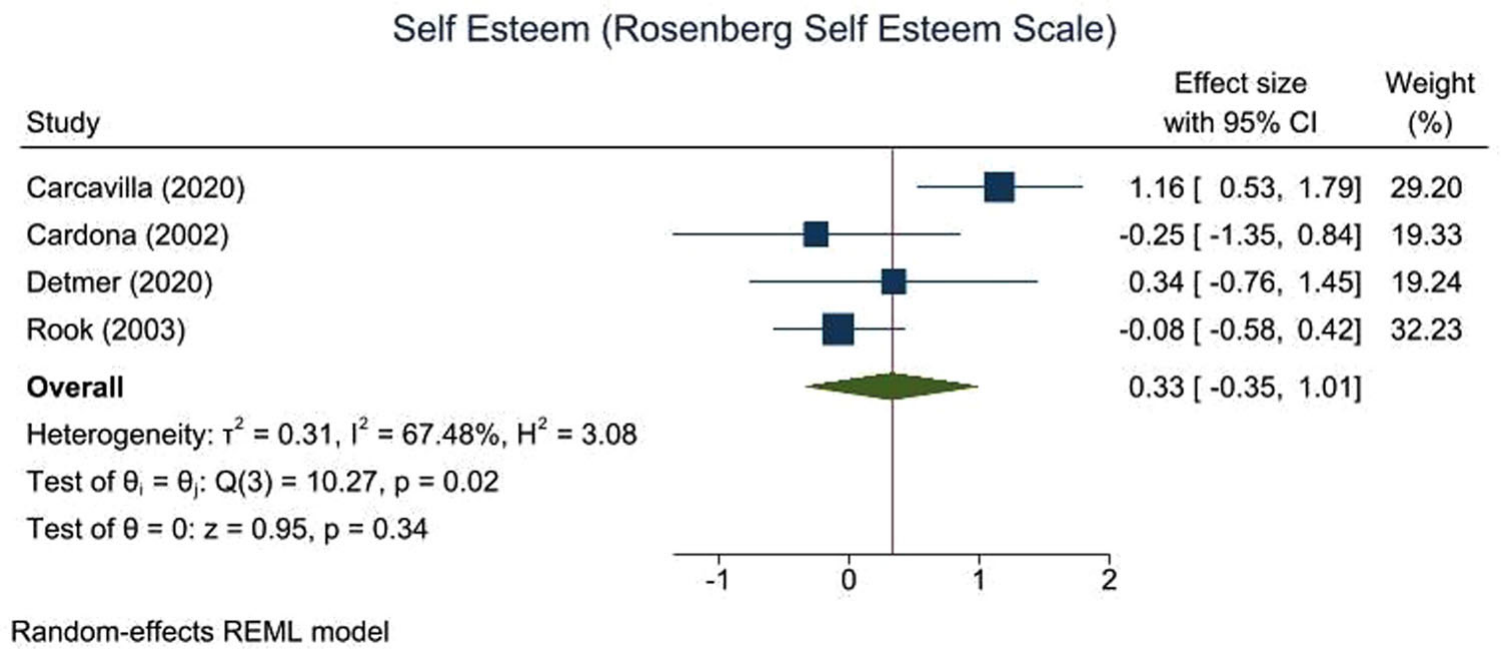
Self‐esteem.

**Figure 6:**
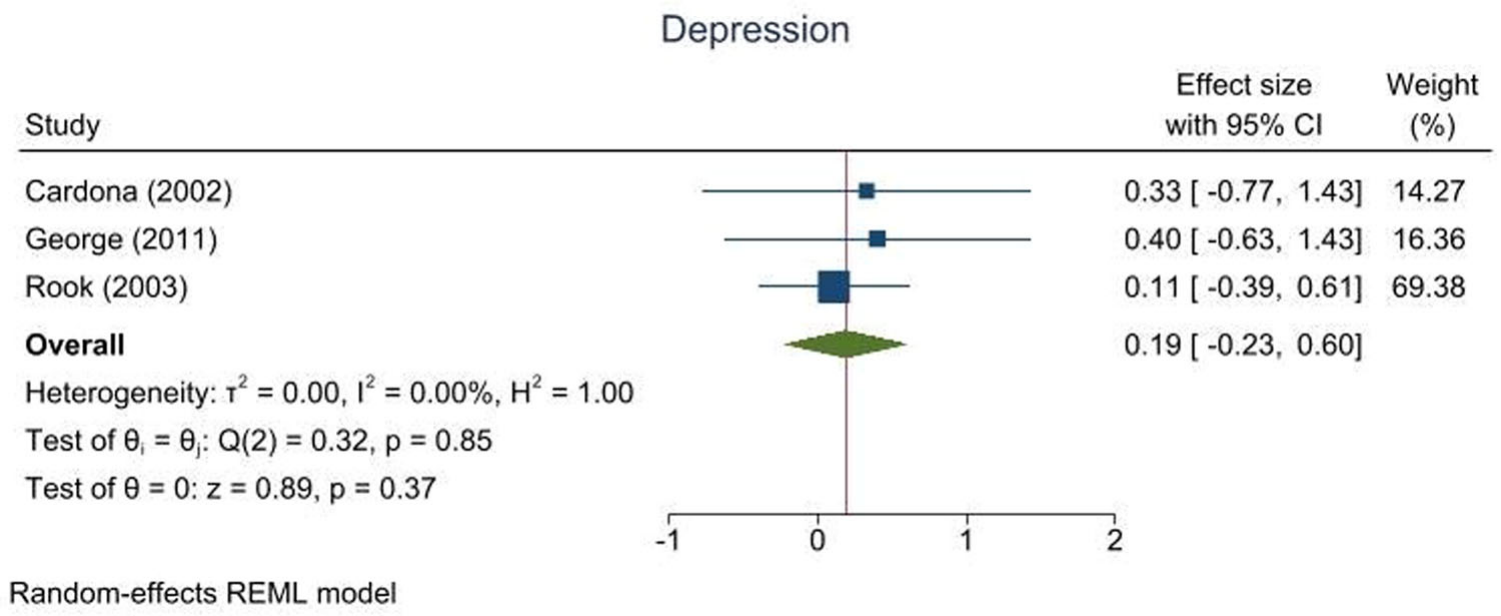
Depression.

From the data collected from these studies we have been able to conduct a meta analysis for the outcomes of self‐esteem (Figure 5) and depression (Figure 6). The interventions consist of different intergenerational activities, had measurements taken at different time points, and with only one study per activity as evidence we cannot imply that these results would be consistent across other studies. Random effects analysis has been used in the two meta‐analyses listed below.

Quality of life, stress, agitation and loneliness were all measured in only one study. Meta‐analysis was therefore not possible; the effectiveness data are presented in Table 5.

**Table 5:** Primary outcomes not in meta‐analysis.

Title	Outcome description	*N*	Timepoint	Outcome type	Intervention	Comparison	ES (95% CI)	SE
Dawson (2017)	Quality of life	17	1 weeks	Continuous	Ageless Play—activity sessions in multigenerational park	Control group—no contact or exercise	‐ Data not available	
Low (2015)	Quality of life	40	1 weeks	Continuous	Grand Friends—nursing home activities with children (Dementia only)	Control group—nursing home activities but no contact	−0.14 (−0.77, 0.49)	0.32
George (2011)	Stress	15	1 weeks	Continuous	Volunteering in school (mentoring and small groups work)	Control group—education sessions only	−1.18 (−2.3, −0.06)	0.57
Low (2015)	Agitation	40	1 weeks	Continuous	Grand Friends—nursing home activities with children (Dementia only)	Control group—nursing home activities but no contact	−1.05 (−1.72, −0.38)	0.34
Rook (2003)	Loneliness	128	1 year	Continuous	Foster Grandparent program (visiting a child in hospital)	Control group—no contact	0.09 (−0.4, 0.58)	0.25

Abbreviations: 95% CI, 95% confidence interval; ES, effect size; *N*, sample size; SE, standard error.

The results of these studies suggest no effect or even some small negative effects (quality of life and loneliness) in older people taking part in intergenerational interventions.

##### Self‐esteem

The results for self‐esteem across four studies (*n* = 254 older people) suggest a non‐statistically significant (ES: 0.33, 95% CI: −0.35, 1.01, *I* ^2^: 67.5%) trend towards small improvements in self‐esteem for the older adults participating in an intergenerational intervention. The studies all used the Rosenberg Self Esteem Scale but measured the outcome at different time points. It is difficult to say if this change is clinically meaningful, but from a public health perspective at a general population level an effect size of 0.2 is considered a small and an effect size at 0.5 is considered a medium but meaningful change. Although the interventions were all Level 5 on the Depth of Engagement Scale (Kaplan, 2004) they involved a wide range of intergenerational activities. For example, one was a language learning activity with adolescents run online over 6 weeks, self‐esteem was measured at 2 weeks postintervention (Carcavilla, 2020), one was a foster grandparent programme where older people visited children with long‐term health conditions in hospital over 3 years, self‐esteem was measured at 1 year postintervention (Rook, 2003), and two were visiting programmes—one based around music with pre‐school children over 12 weeks Detmer 2020 and one based around specific joint projects (art, music or exercises) with older children over 3 weeks (Cardona, 2002), both with self‐esteem measured at 1 week post intervention.

##### Depression

The results for depression across three studies (*n* = 208 older people) suggest little or no impact (ES: 0.19, 95% CI: −0.23, 0.60, *I* ^2^: 0%), with a range of intergenerational activities (though again all Level 5 on the Depth of Engagement Scale) (Kaplan, 2004) presented. It is difficult to say if this change is clinically meaningful, in one study (Cardona, 2002) the change in the intervention group was such that the older people went from reporting moderate levels of depression to mild levels of depression which might be considered meaningful, but their result did not differ significantly from the control group. From a public health perspective an effect size of 0.2 at a general population level is considered a small but meaningful change. Depression was measured using different tools and time points across the studies the Geriatric depression scale at 1 week post intervention (Cardona, 2002); the Beck Depression Inventory at 1 week post intervention (George, 2011); and the Centre for Epidemiological Studies‐Depression scale at 1 year post intervention (Rook, 2003). One was a language learning activity with adolescents run online over 6 weeks (Cardona, 2002), one was a foster grandparent programme where older people visited children with long‐term health conditions in hospital over 3 years (Rook, 2003), and one was an Intergenerational school with mixed ages of children running over 5 months (George, 2011). Although there is a trend towards some positive impacts on depression across the studies the results (both individually and collectively) are not statistically significant.

##### Secondary outcomes

The following 12 outcomes were reported in at least one study: positive and negative affect, agency, self‐efficacy, self‐worth, enjoyment, personal growth, behaviour (intergenerational interactions and engagement), cognitive activity, pleasure, sadness, community, and social activity. Indicators of mental health and wellbeing such as spiritual health and sense of community were not reported in any of the included studies. Due to the inconsistency in data and outcomes, meta‐analysis was considered inappropriate.

We grouped the reported outcomes into summary themes: personal growth, cognitive function, community, affect and engagement/interaction. Groupings were developed and checked with stakeholders who broadly agreed with the names and groupings. The name ‘Personal growth’ for the first group of outcomes was reviewed a number of times, but we ultimately agreed to keep it as it was helpful to highlight that older people can benefit from personal growth too.

##### Personal growth

The outcomes included in this theme reflect concepts around personal growth or understanding of self. Seven studies measured 12 outcomes associated with aspects of personal growth, with effect sizes ranging from 0.18 (small) to 0.80 (large) providing some preliminary evidence that intergenerational interventions might have a positive effect on aspects of a person's sense of self. The results suggest that a person's sense of worth or utility or productivity can be increased by participating in an intergenerational intervention whether that be reminiscing and sharing stories with younger people (Chippendale, 2015), volunteering in schools more generally (George, 2011; Gruenewald, 2016) or working on specific tasks with children (Sipsas‐Herrmann, 2000; Thornton, 2017) (Table 6).

**Table 6:** Personal growth.

Title	Outcome description	*N*	Timepoint	Outcome type	Intervention	Comparison	ES (95% CI)	SE
Chippendale (2015)	Agency	39	1 weeks	Continuous	Living Legends—reminiscence and sharing	Control group—writing self‐story but no sharing	0.63 (−0.02, 1.28)	0.33
Dawson (2017)	Self efficacy	17		Continuous	Ageless Play—activity sessions in multigenerational park	Control group—no contact or exercise	‐ Data unavailable	
	Self worth	17		Continuous	Ageless Play—activity sessions in multigenerational park	Control group— no contact or exercise	‐ Data unavailable	
	Personal growth	17		Continuous	Ageless Play—activity sessions in multigenerational park	Control group—no contact or exercise	‐ Data unavailable	
George (2011)	Sense of purpose	15	1 weeks	Continuous	Volunteering in school (mentoring and small groups work)	Control group—education sessions only	0.76 (−0.3, 1.82)	0.54
	Sense of usefulness	15	1 weeks	Continuous	Volunteering in school (mentoring and small groups work)	Control group—education sessions only	0.32 (−0.7, 1.34)	0.52
Gruenewald (2016)	Generative desire	589	4 months	Continuous	Experience Corps—volunteering in schools	Control group—volunteering but with less potential for intergenerational interaction	0.18 (0.02, 0.34)	0.08
	Generative achievement	589	4 months	Continuous	Experience Corps—volunteering in schools	Control group—volunteering but with less potential for intergenerational interaction	0.29 (0.13, 0.45)	0.08
Shkilnyk (1984)	Life satisfaction	50	1 weeks	Continuous	Nursing home visiting programme	Control group—no visiting	0.44 (−0.13, 1.01)	0.29
Sipsas‐Herrmann (2000)	Generativity	60	1 weeks	Continuous	SCARE (Student Created Aggression Replacement Education)—aggression reduction program delivered by older people	Control group—vocational development program delivered by video	0.80 (0.29, 1.31)	0.26
	Ego integrity	60	1 weeks	Continuous	SCARE (Student Created Aggression Replacement Education)—aggression reduction program delivered by older people	Control group—vocational development program delivered by video	0.27 (−0.24, 0.78)	0.26
Thornton (2018)	Self efficacy for advocacy	50	1 weeks	Continuous	Senior change makers—advocacy program	Control group—physical activity program (less intergenerational contact)	0.42 (−0.17, 1.01)	0.30

Abbreviations: 95% CI, 95% confidence interval; ES, effect size; *N*, sample size; SE, standard error.

##### Cognitive function

Cognitive function was measured in two studies (George, 2011; Sakuri, 2018) using the MMSE (Mini Mental State Examination) typically used to assess cognitive decline. In Table 7 we can see medium to large effect sizes indicating a cognitive benefit for those participating in an intergenerational intervention versus those taking part in an intervention without an intergenerational element. One study even finds positive impact on cognitive function after 6 years of the intervention (Sakuri, 2018)—suggesting potentially lasting effects at least for that particular intergenerational reading intervention (where older adults read picture books to school children aged 4–11years).

**Table 7:** Cognitive function.

Title	Outcome description	*N*	Timepoint	Outcome type	Intervention	Comparison	ES (95% CI)	SE
George (2011)	Cognitive function	15	1 weeks	Continuous	Volunteering in school (mentoring and small groups work)	Control group—education sessions only	0.57 (−0.47, 1.61)	0.53
Sakurai (2018)	Cognitive function	59	6 years	Continuous	REPRINTS—volunteer in school reading program	Control group—no volunteering	0.81 (0.22, 1.4)	0.30

Abbreviations: 95% CI, 95% confidence interval; ES, effect size; *N*, sample size; SE, standard error.

##### Community

Outcomes centring around the impact of intergenerational interventions on the community were lacking with only two studies measuring community related aspects (Table 8). One study (Low, 2015) reported a reduction in the Brief Sense of Community Scale in older adults involved in an intergenerational intervention that involved children visiting a nursing home. The Brief Sense of Community Scale measures psychological sense of community such as group membership and shared emotional connection. In comparison, the other study (Rook, 2003) reported a large positive effect on the number of new relationships gained by those participating in that intergenerational intervention. Although the number of new relationships indicates an increasing community for an individual it may not reflect a ‘sense’ of community. Although both interventions involved children visiting older adults (one in a nursing home [Low, 2015] and the other in a hospital [Rook, 2003]), there are many possible reasons for the difference in findings, for example, the size of the sample (*N* = 40 vs. 108), the outcome measure used, the participant characteristics (older people vs. older people living with dementia) or the time point (1 week vs. 1 year).

**Table 8:** Community.

Title	Outcome description	*N*	Timepoint	Outcome type	Intervention	Comparison	ES (95% CI)	SE
Low (2015)	Sense of community	40	1 weeks	Continuous	Grand Friends—nursing home activities with children (Dementia only)	Control group—nursing home activities but no contact	−0.28 (−0.91, 0.35)	0.32
Rook (2003)	Number of new relationships	128	1 years	Continuous	Foster Grandparent program (visiting a child in hospital)	Control group—no contact	1.34 (0.79, 1.89)	0.28

Abbreviations: 95% CI, 95% confidence interval; ES, effect size; *N*, sample size; SE, standard error.

##### Affect

The theme ‘Affect’ reflects various elements of emotion, five aspects of affect were measured across three studies (Carcavilla, 2020; Dawson, 2017; Low, 2015). In Table 9 we can see that the effect sizes range from 0 to 0.64 (medium positive effect). Interestingly, the two studies we have data for both indicate a small‐medium positive effect on positive emotions such as pleasure, and both report either no effect or a positive effect on negative emotions (i.e., reduce level of negative emotions in the intervention group [Carcavilla, 2020]). However, the interventions were different (one an online interaction for language practice [Carcavilla, 2020], the other a visiting programme in a nursing home [Low, 2015]) and engaged different populations (one with teenagers in education and older adults in a care home [Carcavilla, 2020], the other with pre‐schoolers and older people living with dementia [Low, 2015]).

**Table 9:** Affect.

Title	Outcome description	*N*	Timepoint	Outcome type	Intervention	Comparison	ES (95%CI)	SE
Carcavilla (2020)	Positive affect	46	2 weeks	Continuous	Smile Connect Online contact to help language development	Language practice in school only	0.30 (−0.29, 0.89)	0.30
	Negative affect	46	2 weeks	Continuous	Smile Connect Online contact to help language development	Language practice in school only	−0.58 (−1.17, 0.01)	0.30
Dawson (2017)	Enjoyment	17		Continuous	Ageless Play—activity sessions in multigenerational park	Control group—no contact or exercise	‐ Data unavailable	
Low (2015)	Pleasure	40	1 weeks	Continuous	Grand Friends—nursing home activities with children (Dementia only)	Control group—nursing home activities but no contact	0.64 (−0.01, 1.29)	0.33
	Sadness	40	1 weeks	Continuous	Grand Friends—nursing home activities with children (Dementia only)	Control group—nursing home activities but no contact	0 (−0.63, 0.63)	0.32

Abbreviations: 95% CI, 95% confidence interval; ES, effect size; *N*, sample size; SE, standard error.

##### Engagement/interaction

A total of 12 different outcomes related to engagement in activities and intergenerational interactions were measured by four studies (Detmer, 2020; Giglio, 2006; Low, 2015; Shkilnyk, 1984). One study reports intergenerational interactions (Giglio, 2006) using eight measures including verbal interaction, spontaneous touching, spontaneous hand holding and spontaneous hugging, both during and shortly after a music intervention. Effect sizes range from 0.50 (medium effect) to 3.32 (large effect) although these results are from a small sample size. The results for level of engagement presented in Table 10 are a little more diverse with some positive trends and some negative—this may reflect the level of engagement required by an intervention or a change in activity or the way engagement is measured in a particular study. For example, active engagement appears to be lower than the control group in the final session of Grand Friends (Low, 2015) whereas in previous weeks it had been higher than the control group—the authors suggest this is due to a change in the activity in the final week which was a party—the usual structured activities were not in place.

**Table 10:** Engagement/interaction.

Title	Outcome description	*N*	Timepoint	Outcome type	Intervention	Comparison	ES (95% CI)	SE
Detmer (2020)	Intergenerational interactions	13		Continuous	Music therapy	Wait‐list control—no contact	‐ Data unavailable	
Giglio (2006)	Spontaneous smiling	29	1 weeks	Continuous	Music therapy	Control group—no music or interaction	1.20 (0.2, 2.2)	0.51
	Spontaneous laughing	29	1 weeks	Continuous	Music therapy	Control group—no music or interaction	0.23 (−0.67, 1.13)	0.46
	Spontaneous verbal interaction	29	1 weeks	Continuous	Music therapy	Control group—no music or interaction	0.50 (−0.42, 1.42)	0.47
	Spontaneous head nodding	29	1 weeks	Continuous	Music therapy	Control group—no music or interaction	0.25 (−0.65, 1.15)	0.46
	Spontaneous touching	29	1 weeks	Continuous	Music therapy	Control group—no music or interaction	3.32 (1.83, 4.81)	0.76
	Spontaneous hand holding	29	1 weeks	Continuous	Music therapy	Control group—no music or interaction	0.68 (−0.26, 1.62)	0.48
	Spontaneous tapping	29	1 weeks	Continuous	Music therapy	Control group—no music or interaction	−1.66 (−2.74, −0.58)	0.55
	Spontaneous hugging	29	1 weeks	Continuous	Music therapy	Control group—no music or interaction	1.50 (0.46, 2.54)	0.53
Low (2015)	Active engagement	40	1 weeks	Continuous	Grand Friends—nursing home activities with children (Dementia only)	Control group—nursing home activities but no contact	−0.59 (−1.22, 0.04)	0.32
	Passive engagement	40	1 weeks	Continuous	Grand Friends—nursing home activities with children (Dementia only)	Control group—nursing home activities but no contact	0.77 (0.12, 1.42)	0.33
Shkilnyk (1984)	Social activity	50	1 weeks	Continuous	Nursing home visiting programme	Control group—no visiting	0.18 (−0.39, 0.75)	0.29
	Dyad interactions	50		Continuous	Nursing home visiting programme	Control group—no visiting	Data unavailable	

*Note*: NB spontaneous tapping is a negative behaviour outcome the Effect size reported here suggests it is reduced with the intervention.

Abbreviations: 95% CI, 95% confidence interval; ES, effect size; *N*, sample size; SE, standard error.

Data on intergenerational interactions (or dyads) is missing from two studies (Detmer, 2020; Shkilnyk, 1984)—these interactions were observed over time but not compared to the control group within their studies. Both studies report an increase in intergenerational interactions over time.

*Research Question 2: What characteristics of intergenerational activities are associated with a positive impact on the wellbeing and mental health of older people?*

To address Research Question 2 we planned to use information on intervention characteristics such as setting, context, intensity, duration etc. However, due to the small number of eligible studies, and the variation in interventions and outcomes it has not been possible to determine which intervention characteristics are associated with a positive impact on the wellbeing and mental health of older people.

*Research Question 3: What are the underlying theories for the effectiveness of intergenerational activities in older people?*

In the literature regarding the theories behind intergenerational interventions several theories are highlighted, some more common than others (Jarrott, 2011; Kuenhe, 2014).

Many of the studies do not explicitly refer to named theories that have informed the development or logic of the intervention but use language or logic that reflects the notion or sentiment of relevant theories. Table 11 documents each intervention, its aim and the theories that are implicitly or explicitly cited within the papers. The most commonly reported (*named*) theory is Erikson's Theory of Psychosocial Development which was explicitly mentioned in three studies (Chippendale, 2015; Dawson, 2017; Sipsas‐Herrmann, 2000). Kuenhe (2014) states ‘More specifically, it has been suggested that Erikson's seventh stage of psychosocial development, generativity versus stagnation, fits well with an intergenerational approach Kuenhe 2014. According to Erikson, generativity involves perceiving one's self as connected with a future that will survive and continue after one is gone, giving of self to the future, and a hope that the future is secure’ (Erikson, 1982 as cited in Kuenhe 2014). However, the most common theory *implied* by intervention logic descriptions is Contact Theory which was identified in 12 of the 14 intervention descriptions. Again, described by Kuenhe (2014) Contact Theory states ‘social contact between segregated groups can facilitate more accurate perceptions and reductions in prejudice, but suggest this occurs only under certain conditions….’ (Allport, 1954 as cited by Kuenhe, 2014) specifies four key conditions necessary for optimal contact: equal group status within the situation; common goals; intergroup cooperation; and the support of authorities, law, or custom (Kuenhe, 2014).

**Table 11:** Implicit and explicit program theories.

Item	Intervention name	Intervention aim	Intergenerational Theory (implicit or explicit)	Associated theories
Carcavilla (2020)	Smile Connect	The aim of this intervention promotes intergenerational contact between young adults in secondary schools in Italy and older adults in care homes in Spain. The purpose was to examine the effectiveness of a Spanish language educational videoconferencing programme between generations, on the one hand in reducing negative attitudes towards ageing and improving emotional affect among young adults and on the other hand, for improving emotional affect and self‐esteem among older adults.	Implicit	Contact theory implied ‘Our study promotes intergenerational contact between young adults in secondary schools in Italy and older adults in care homes in Spain’.
Cardona (2002)	None**—**Task orientated intergenerational program	The aim of this intervention is to use a task‐oriented approach to intergenerational program for teenagers and older adults. It is believed this program may allow positive influences between both groups and may help both groups to increase or improve their sense of self‐efficacy, self‐esteem and depression secondary to being able to accomplish a goal.	Implicit	Contact theory implied Task‐orientated approach ‘Social interactions and influences are often the foundation for the formulation of each individual's identity. Interactions between adolescents and older adults can benefit both groups. The implementation of an after school task oriented intergenerational program may allow positive influences between both groups and may help both groups to increase or improve their sense of self‐efficacy secondary to being able to accomplish a goal and being able to work on a specific and structured task’.
Chippendale (2015)	Living Legends	The aim of this intervention is that older adults who participate in Living Legends volunteer programme would have an enhanced sense of purpose and meaning in life compared with older adults who participated in life review writing alone. Volunteering to work on a task together is believed to enhance mental wellbeing and life satisfaction through an improved sense of purpose.	Explicit	Erikson's theory of Psychosocial Development (human development includes mentoring the next generation and reflecting back on one's life as a whole) Contact theory also implied ‘Therapeutic benefits of volunteer programs that incorporate an intergenerational exchange include enhanced well‐being (Yuen, 2008), increased intergenerational understanding (Underwood, 2006; Zucchero, 2010), appreciation of the opportunity to share stories (Chonody, 2013), opportunity to serve as a role model and mentor and form mutual relationships (Zucchero, 2010), and decreased depressive symptoms (Chung, 2009). Given that both life review through writing and intergenerational programs offer therapeutic benefits for older adults, combining the two interventions may multiply their benefits. In addition to addressing depressive symptoms through life review, the combined approach can target sense of purpose and meaning in life, a factor known to mitigate functional decline. This combined approach is consistent with Erikson's (1950) Theory of Psychosocial Development, in which the last two stages of human development include mentoring the next generation and reflecting back on one's life as a whole’.
Dawson (2017)	Ageless Play	The aim of this intervention is that that users (primary school aged children and older people) of multi‐generational play parks integrated with stealth exercise will experience a boost in energy and reap benefits of fresh air and nature while undergoing simple recreational and leisure activities. The theory suggests that playing with children allows older adults an opportunity to reminisce about their past childhood, while children receive an enriched learning experience from interacting with positive role models, and this also supports the concept of active ageing.	Explicit	Erikson's theory of Psychosocial Development Theory of planned behaviour Contact theory implied ‘Significant benefits provided by intergenerational activities for older adults are (1) the experiences that come with it can be ideal for older adults to prevent and resolve issues that occur in late life, and (2) intergenerational activities that are designed to help youth successfully assist older adults in accomplishing certain life stages outlined by Erikson, such as integrity versus despair. Evidence also shows that playing with children allows older adults an opportunity to reminisce about their past childhood, while children receive an enriched learning experience from interacting with positive role models. … intergenerational programming at a multi‐ generational play park is highly likely to foster interaction, teamwork, and relationship building between older adults and children’.
Detmer (2020)	None (Intergenerational music therapy)	The aim of this intervention was to identify the effects of an intergenerational music therapy program on children's literacy, older adults’ physical functioning and self‐worth, and interactions between the two age groups.	Implicit	Contact theory implied Music therapy to aid language development and to aid exercise ‘Overall, the literature on intergenerational programming has been primarily focused on improving cross‐age attitudes, interaction, and quality of life mea‐sures……Music therapy is an evidence‐based health‐care profession that uses music to improve non‐musical goals such as academic skills, communication, motor ability, and social‐emotional functioning’.
George (2011)	None (Intergenerational Volunteering)	The Intergenerational School is structured around the ideology that people of all ages can learn alongside each other throughout their life spans, including those in the long‐term care community—some with memory loss. The aim is to see if volunteering as a mentor in TIS impacts depression, anxiety, quality of life, agency, Self‐efficacy and cognitive activity in older people.	Implicit	Contact theory implied Some theory around volunteering ‘A subset of research has established that older adults who form relationships with children through intergenerational volunteering programs seem to experience specific benefits, such as improvements in health status and well‐being, increased activity, strength, and cognitive ability, the creation of meaningful relationships, enhanced self‐ esteem, increased social capital, and better psychological functioning. The Intergenerational School is structured around the ideology that people of all ages can learn alongside each other throughout their life spans’.
Giglio (2006)	None	This intervention aimed to examine the effect of a music therapy intergenerational program between pre‐school children and older adults with dementia on cued and spontaneous behaviours of the older adults. The intervention intends to boost social support and connections to aid older adults to remain engaged and therefore improve their wellbeing, life satisfaction and self‐esteem.	Explicit	Music therapy to aid language development and memory Disengagement theory Socioemotional Selectivity Theory Activity Theory Research in music therapy in the geriatric field has shown that many different music therapy activities have been beneficial with working with older adults with dementia. …Singing evokes the use of memory with recalling the words or familiar melody of a song, and may trigger remembering where, when and/or who sung the song to them…’. ‘Both the activity and disengagement theories relate to the socioemotional selectivity theory. As one ages, emotionally based social relationships become more important. Older adults therefore disengage from other people in society that they are not as emotionally attached to and begin to actively pursue more emotionally close intergenerational relationships with family and close peer relationships with long‐time friends (Baltes, 1999).’
Gruenewald (2016)	Experience Corps	This intervention aims to attract older adult participants through the opportunity for generative engagement, then via cognitive, physical, and psychosocial pathways to enhance the health and well‐being of older adult volunteers while simultaneously promoting the academic and psychosocial well‐being of elementary schoolchildren and the climate and social capital of the school.	Implicit	Evidence‐based health promotion Civic engagement program designed to harness the time, energy, and wisdom of older adults to improve academic outcomes of elementary school children. ‘EC is designed to attract older adult participants through the opportunity for generative engagement and then to operate via cognitive, physical, and psychosocial pathways to enhance the health and well‐being of older adult volunteers while simultaneously promoting the academic and psycho‐social well‐being of elementary schoolchildren and the climate and social capital of the school and community in which the EC program resides’.
Low (2015)	Grandfriends	This intervention aims to be enjoyable, encourage interaction, and develop relationships between the generations by encouraging both groups to work together towards a common goal. It is hoped that increased engagement during the activity will better meet needs for meaningful activity and social engagement and will lead to improvements in quality of life and sense of community and in decreased agitation among those with dementia symptoms.	Implicit	Contact theory implied Task‐orientated approach ‘Intergenerational programs bring together older adults and children or adolescents to participate in a shared activity. These have been shown to have benefits for the older participants such as improved depression and quality of life… It has been argued that intergenerational programs that provide exposure to but only minimal interaction with older adults and without planned curricula may result in a decrease in children's positive attitudes towards ageing and older people. Alternatively programs with higher quality, frequency, and duration of intergenerational interactions may be more likely to cultivate positive attitudes (Femia, 2008)’.
Rook (2003)	Foster Grandparent program	This intervention aimed to involve older adults in a social role to improve their psychological well‐being (psychological health and self‐worth) through activities that were expected to facilitate the formation of new social ties and by providing a context in which participants regularly helped to nurture and care for a developmentally‐disabled child.	Implicit	Contact theory implied (social engagement) ‘We anticipated that such regular contact, organised around shared activities, would facilitate the acquaintanceship process. Moreover, the program involved frequent contact extended over a sufficiently long period of time to allow such relationships to emerge gradually and in the relatively natural and familiar context of shared activities’.
Sakurai (2018)	REPRINTS	This intervention engages older adults in reading picture‐books to kindergarten and elementary school students, with the expectation that it will help maintain or improve the cognitive and physical functions of older adults. The program is expected to establish new social networks with members, children, teachers, and program staff, and contribute to the healthy upbringing of children.	Implicit	Social engagement/socio cultural theory implied? ‘Social engagement decreases the risk of cognitive impairment and incident dementia… Therefore, social engagement programs may be considered effective and sustainable sources of cognitive and physical exercises and can be implemented in community‐based settings to improve cognitive abilities…. The program is expected to establish new social networks with members, children, teachers, and program staff, and contribute to the healthy upbringing of children’.
Shkilnyk (1984)	None (type of visiting programme)	This intervention aims to engage older people and adolescents in a social intervention program in the belief that social engagement is good for health and life satisfaction (engagement or companionship) in older adults and attitude change and formation in adolescents.	Explicit	Disengagement theory ‘The disengagement model emphasises the synchrony of timing of social and individual changes. Thus, as society reduces activities for the aged, there is a concomitant reduction in role involvement for the aged’.
Sipsas‐Herrmann (2000)	SCARE (Student Created Aggression Replacement Education)	This intervention aims to (a) teach young people about emotions, including aggression and anger, (b) help young people recognise alternatives to violent behaviour and aggressive responses, and (c) encourage youth to make good decisions in response to provocative situations. This programme is delivered by older people as trainers (rather than student trainers) in the belief that the life experiences of the older people will help to change attitudes towards older people and perhaps that of older people towards younger people as well as their life satisfaction and mental wellbeing.	Explicit	Erikson's Theory of psychosocial development Contact theory implied? Includes other theories around anger management ‘According to Herrmann and McWhirter, the treatment package is based on the tenet that angry and aggressive individuals hold biased, hostile attributions or beliefs about the intentions of others. The SCARE program perspective is that teaching reattribution of perceived offenses and the control of resulting anger is key to preventing violent and aggressive acts from occurring’. ‘From a theoretical perspective, Erik Erikson's theory of human development best captures why older adults benefit from intergenerational programs. Erikson (1950/1963) believed that development continues throughout a person's entire lifetime’. ‘For the older adults, intergenerational contact has been shown in various studies to: (a) improve physical, cognitive and emotional functioning (Allis, 1989); (b) increase morale and feelings of self‐worth (Dellmann‐Jenkins, 1997; Midlarsky, 1994; ReVille, 1989); and (c) increase life satisfaction (McCrea, 1997)’.
Thornton (2018)	Senior Change makers	The aim of this intervention was to improve advocacy skills and confidence in older people through an intergenerational community building/advocacy program with college students.	Explicit	Social‐cognitive theory Empowerment theory Contact theory implied Task orientated ‘At the individual level, the intervention applied Social Cognitive Theory by providing participants training and opportunities to develop advocacy skills and confidence (Table 1). Empowerment Theory involves enabling community members to take control of their lives and their environments (Zimmerman, 1988). … The empowerment process allows disadvantaged people to gain greater access to and control over community resources (Lawrence‐Jacobsen, 2005). … At the social level, Contact Theory was applied to guide intergenerational interactions. Contact Theory posits that certain types of interactions between generations can reduce ageism and help members of different generations develop informed perceptions of each other (Kuehne & Melville, 2014)'.

As each intervention is, by its nature, complex it isn't unexpected to find multiple theories discussed within one intervention. It is concerning that named theories are not more evident in the literature—this suggests that those developing/testing interventions have not properly considered the underlying theory of change.

## 6 DISCUSSION

### 6.1 Summary of main results

This systematic review found 14 randomised controlled trials looking at the impact of intergenerational interventions on the mental health and wellbeing of older people. The quality of the trials and the length of follow‐up is poor as is the reporting of equity characteristics. Many relevant outcomes have been studied but often with very little overlap across studies. The exceptions to this are the outcomes of self‐esteem and depression which have been measured in three or more studies. The effect size for self‐esteem indicates a small positive impact, the effect size for depression indicates little/no impact, but the results are not certain due to the small samples sizes and few studies available. The lack of overlap of outcomes and the lack of studies on similar interventions or interventions with similar elements means it is difficult to determine if any, one intervention or intervention characteristic is more or less effective for any given outcome. The primary objectives of many of these studies was not to influence wellbeing, nevertheless, there is some indication that wellbeing was improved. There are likely to be many factors that will influence participants wellbeing as a result of participating in these types of interventions (Jarrott, 2021).

However, this information is useful as it can help us to begin to understand if an intervention isn't appropriate for a particular setting, population activity or to achieve a particular outcome. For example, Grand Friends (Low, 2015) is an intergenerational intervention where young children visit older people living with dementia in their care home. The results we have been able to report for this study suggest that whilst this intervention may have been able to reduce levels of agitation and increase some reports of pleasure, it did not have the same beneficial effects on quality of life, sadness or improvements in engagement. The rationale for this intervention was that increased engagement during the activity would meet needs for meaningful activity and social engagement and result in improvements in quality of life and sense of community and in decreased agitation amongst those with dementia symptoms who participate in Grand friends in comparison to a control group with no interaction. This may indicate that whilst some of the desired outcomes were achieved—others were not, and that perhaps engagement during an activity was not the method by which this intervention works, or perhaps the activities set were not appropriate to promote the right level of engagement.

### 6.2 Overall completeness and applicability of evidence

Overall, the state of the evidence for intergenerational interventions is patchy with poor methodological quality. Consequently, it is difficult to describe what does and doesn't work to improve mental health and wellbeing outcomes in older adults using intergenerational activities. This is partly explained by the wide variation of interventions and intervention elements and characteristics. Although, as we see here, there are studies using randomised controlled trial designs—due to the complex nature of intergenerational interventions conducting studies of this kind is complicated and costly and often outcomes are only measured after a short follow‐up period. This means that there are very few studies of effectiveness of these interventions, and we can't be confident of what the effects of intergenerational interventions are in older people. From the research presented here there are many gaps which still need to be filled. We need to understand much more about the different elements of interventions as well as the interventions as a whole, and we need to better understand what individual and community outcomes can be influenced by these and how. To date there is not enough information to have good summary level evidence of effectiveness. These gaps illustrate the challenges of standardising curricula and programming and being able to generalise findings across the natural variation of intergenerational interventions.

Whilst the theories identified in some of the included studies are described in the intergenerational literature more generally, many studies lacked detail of the named theories underlying the intervention.

### 6.3 Quality of the evidence

The overall quality of the evidence is poor. The most limiting factor being the blinding of participants, personnel and outcome assessment, allocation concealment and sample size. Although these trials may be demonstration projects testing to see if something works or not most failed to report power calculations to ensure the appropriate sample size was achieved. We also saw that the outcomes measured were so variable that a lot of the research could not be brought together in meta‐analyses. More consistent and agreed measure for reporting outcomes would benefit future research in this area. We also noted that despite the intervention involving two groups of people, in some studies the younger generation were considered part of the intervention and so the impact of being involved in the intervention on them was not measured. This is a serious ethical consideration both in terms of participation of research and research waste.

### 6.4 Equity

We used the progress plus framework (O'Neill, 2014) to establish what information and characteristics were captured and/or targeted in this body of evidence. In summary, many of the equity characteristics were not reported. Commonly but inconsistently, reported characteristics of the populations involved in the studies were gender and race/ethnicity, with some reporting on levels of education and socioeconomic status, other personal characteristics that were commonly reported were cognitive decline and physical health impairments. However, although these characteristics are recorded they are not necessarily accounted for in the analysis or subgroup analysis of the results.

### 6.5 Potential biases in the review process

In this review we decided to only include randomised controlled trials as they provide the best evidence to address effectiveness questions and the EGM suggested there were sufficient studies available. However, due to the variability in the types of intergenerational intervention and the elements they consist of it is possible we are missing out on information that other study designs could have provided to further inform research questions 2 and 3. Future research should consider what the best study design might be and what information it is important to capture and how long for. Intergenerational interventions are by nature complex, so they need funding for the best and most informative research to be conducted, for example, pragmatic trial designs developed to evaluate complex social interventions may be applicable.

It is also interesting to note that most of the interventions included in the review were categorised as level 5 interventions (on the Depth of Engagement scale [Kaplan, 2004])—these are ongoing intergenerational activities over a defined period of time and are often implemented on an experimental or trial basis, and frequently depend on external funding. This is perhaps something that might be a construct of how long and for what research funding is provided. The impact is that we are missing evidence from trials that measure outcomes on a longer term basis and that many interventions only last while the research funding does which means the efforts behind setting up the intervention are lost along with the relationships that have formed and any other potential benefits.

The themes used in describing the outcomes were named by RW in an attempt to best capture and group the outcomes that were reported, these are the groupings and names we found helpful in this work. However, they could be re‐grouped and re‐named from another perspective.

### 6.6 Agreements and disagreements with other studies or reviews

In comparison to a similar review conducted in 2021 (Krzeczkowska, 2021) we found nine more randomised controlled trials in this area. However, our conclusions about the need for more research of a better standard is in agreement with theirs. Their review incorporates broader study designs, and although they suggest general trends and positive benefits on outcomes these can also not yet be formally concluded.

Similarly to Jarrott (2021) we noted the often small sample sizes, the need for more rigorous evaluation and the need to include outcomes measures for younger generation participants as well as parents and carers. Over the last 20 years several (~10) theories have been identified as informing the development of intergenerational interventions (Jarrott, 2011; Kuenhe, 2014). In 2014 Kuehne (Kuenhe, 2014) found that over time the use of theories in intervention development is increasing and that of the 10 theories identified Contact theory and Erikson's theory of psychosocial development are the most commonly reported theories along with the Theory of personhood. Similarly, in the studies included in our review we found Erikson's theory of psychosocial development to be the most common *named* theory and Contact theory the most commonly implied theory (though the latter is subject to reviewer bias). Whilst not many of the ~10 theories previously highlighted in the literature could be identified in the included studies in this review it is possible that elements of them and other theories are present, just not obviously identifiable. However, newly developed interventions would benefit from taking account of known theories and how they can influence the content, structure and outcomes of the interventions they intend to provide.

## 7 AUTHORS’ CONCLUSIONS

### 7.1 Implications for practice and policy

Intergenerational interventions show some promise but lack sufficient research across the variety of interventions and outcomes means we are unclear what their potential may be.

Plans for intervention sustainability would benefit any effective interventions (suggested or existing).

Commissioners and intervention developers should ensure interventions provide sufficient theoretical evidence for the logic behind the proposed intervention.

Commissioners and intervention developers should improve their consideration of equity within the interventions.

More understanding is needed on how best to measure community related outcomes and what is really meant by this.

### 7.2 Implications for research

Research on intergenerational interventions needs more consistent and agreed measures for reporting individual outcomes and community outcomes (core outcome sets).

More understanding is needed on how best to measure ‘community’ outcomes.

Trialists should be performing power calculations to adequately power studies to understand how interventions may impact different members of society differently (equity) and how any impact remains for the long term.

Research methods would benefit from establishing outcomes for a given population from a variety of perspectives to overcome issues of bias from the lack of blinding of measures recorded by self report.

Research on intergenerational interventions should measure outcomes for BOTH the older and younger population engaged in the intervention—these may or may not be the same outcomes reflected in both populations.

Further research is needed on the long term impact of interventions on outcomes (whether participants need to keep being involved in an ‘intervention’ to continue to benefit) and sustainability of interventions beyond the initial funding of the research project—our stakeholders highlighted that interventions that are initiated for research and then end (usually within a year) are not helpful.

## CONTRIBUTIONS OF AUTHORS

- Content: ERC is a practitioner and consultant based in Plymouth and Project Manager at The Sensory Trust where she works on the Creative Spaces in the Community Project. This project uses nature and outdoor spaces to encourage older people with dementia to become more active, build social networks and foster independence. Previously she founded the multi‐award winning Penryn Memory Café and led a memory café in York for 2 years whilst at University. She has recently completed the International Certificate in Intergenerational Practice provided by Generations Working Together and the University of Granada. SC is Commissioning Manager at NHS Kernow Clinical Commissioning Group and has an interest in the role of intergenerational programmes and activities in health and social care. RS is an advanced public health specialist at Cornwall Council with an interest in the role of intergenerational programmes and activities in health and social care specifically in relation to the mental health of older adults. RF is an older man living with dementia who has experience of intergenerational programmes.
- Systematic review methods: JTC is an expert in evidence synthesis and health policy research. She is co‐chair and editor of the Ageing Group of the Campbell Library and co‐director of the Cochrane Campbell Global Ageing Partnership. RW is an expert in evidence synthesis methods. FC is editor of the Children and Adolescent Group of the Campbell Collaboration. She has over 20 years of experience in evidence synthesis. DK is an expert in synthesising evidence for social policy and developing methods to enhance the use of evidence in decision making. GJMT is an expert in evidence synthesis with skills in quantitative and qualitative synthesis methods. RG is an expert in qualitative synthesis methods.
- Statistical analysis: GJMT is an expert in evidence synthesis with skills in quantitative and qualitative synthesis methods.
- Information retrieval: MR is an information specialist with experience in health services research, methods editor for the Ageing Group of the Campbell Library and a member of the Campbell Information Retrieval Methods Group. AS is a Senior Information Specialist, with extensive experience of literature searching and information management for systematic reviews and other types of evidence syntheses on a wide range of topics.

## DECLARATIONS OF INTEREST

ERC, members of our advisory group and members of the Only Connect steering group are involved in the delivery of intergenerational activities and programmes.

## PRELIMINARY TIMEFRAME

We plan to submit the systematic review for peer review in December 2022.

## PLANS FOR UPDATING THIS REVIEW

Once completed the systematic review will be updated as resources permit.

## DIFFERENCES BETWEEN PROTOCOL AND REVIEW

In our protocol we said we would set up automated search alerts to identify additional relevant literature which we will use to update the map as the project progresses; any studies identified by this process will be screened for eligibility in both the map and the review, however this has not yet been completed.

We used the Cochrane Risk of Bias tool instead of ROB2 as the variation in the outcomes reported (and therefore inability to group outcomes for meta‐analysis) was such that the extra level of detail required in the ROB2 seemed disproportionate to the value that it would give for the synthesis required in this review.

As well as extracting information on the theories identified by the authors of the studies we also attempted to identify theories that were implied in the text, though we accept there are weaknesses to this approach.

## PUBLISHED NOTES

**Characteristics of studies**

**Characteristics of included studies**

Carcavilla 2020

**Table:** 

**Notes**
Risk of bias table
Cardona 2002
**Notes**
Risk of bias table
Chippendale 2015
**Notes**
Risk of bias table
Dawson 2017
**Notes**
Risk of bias table
Detmer 2020
**Notes**
Risk of bias table
George 2011
**Notes**
Risk of bias table
Giglio 2006
**Notes**
Risk of bias table
Gruenewald 2016
**Notes**
Risk of bias table
Low 2015
**Notes**
Risk of bias table
Rook 2003
**Notes**
Risk of bias table
Sakuri 2018
**Notes**
Risk of bias table
Shkilnyk 1984
**Notes**
Risk of bias table
Sipsas‐Herrmann 2000
**Notes**
Risk of bias table
Thornton 2017
**Notes**
Risk of bias table
*Footnotes*
Characteristics of excluded studies
Carlson 2008
**Reason for exclusion**
Fried 2004
**Reason for exclusion**
*Footnotes*

**Characteristics of ongoing studies**

***Digital Buddy: Digital Inclusion for the Elderly***

**Table:** 

**Study name**	
**Starting date**	
**Contact information**	Sally Chan
**Notes**	

***INTEGRITY***

**Table:** 

**Study name**	The INTErGenerational intervention taRgeting fraIlTY trial (INTEGRITY)
**Starting date**	
**Contact information**	rpeters@georgeinstitute.org.au
**Notes**	

## SOURCES OF SUPPORT

**Internal sources**

- No sources of support provided
**External sources**
- NIHR, UK

The systematic review is funded by the National Institute for Health Research (NIHR) Evidence Synthesis Programme NIHR 133097 and NIHR 133172 and supported by the National Institute for Health Research (NIHR) Applied Research Collaboration South West Peninsula. The views expressed are those of the author(s) and not necessarily those of the NIHR or the Department of Health and Social Care.

## Supporting information

Supporting information.
