# Supplementary material for: What is the effect of intergenerational activities on the wellbeing and mental health of older people?: A systematic review
Source: Campbell Syst Rev. 2023 Oct 3;19(4):e1355. doi: 10.1002/cl2.1355 (PMC10546258; doi:10.1002/cl2.1355)
Supplement: Supplementary file 1 — Supporting information. [file CL2-19-e1355-s001.docx]

Appendices

1 Appendix A - EGM search strategies

[Enter text here]Database Search Strategies

MEDLINE via OvidSp

Database: Ovid MEDLINE(R) ALL <1946 to July 21, 2021>

Search Strategy:

--------------------------------------------------------------------------------

1 Intergenerational Relations/ (4146)

2 (intergenerational or inter generational).ti,ab. (6528)

3 cross age.ti,ab. (109)

4 across generation*.ti,ab. (2061)

5 cross generation*.ti,ab. (438)

6 ((generations or different age groups or all ages or all age groups or mixed ages or mixed age groups or (old* adj2 young*)) adj5 (together or social engagement or connecting)).ti,ab. (257)

7 (intergenerational adj2 (program* or intervention*)).ti. (71)

8 or/1-6 (11703)

9 adolescent/ or child/ or child, preschool/ (3143466)

10 child*.ti,ab. (1463735)

11 (young adj (person or people or male* or female*)).ti,ab. (54389)

12 (youth* or teen*).ti,ab. (110196)

13 young offender*.ti,ab. (499)

14 (school and pupil*).ti,ab. (4871)

15 preschooler*.ti,ab. (7441)

16 student*.ti,ab. (304578)

17 (girl or girls or boy or boys).ti,ab. (240923)

18 or/9-17 (3888702)

19 exp Aged/ (3276163)

20 dementia.ti,ab. (115820)

21 alzheimer*.ti,ab. (156770)

22 old*.ti,ab. (1566913)

23 elderly.ti,ab. (258523)

24 geriatric.ti,ab. (44411)

25 (residents or resident).ti,ab. (170641)

26 (elder or elders).ti,ab. (16913)

27 (retired or retiree*).ti,ab. (7895)

28 veteran*.ti,ab. (38644)

29 grandfriend*.ti,ab. (0)

30 seniors.ti,ab. (7890)

31 (senior adj citizen*).ti,ab. (1548)

32 (centarian* or centenarian* or nonagenarian* or octagenarian* or octogenarian* or sexagenarian* or septuagenarian*).ti,ab. (6890)

33 or/19-32 (4773325)

34 program*.ti,ab. (937612)

35 activit*.ti,ab. (3230552)

36 interaction*.ti,ab. (1398324)

37 (project or projects).ti,ab. (220965)

38 intervention*.ti,ab. (1099124)

39 initiative*.ti,ab. (95427)

40 scheme.ti,ab. (104838)

41 visit*.ti,ab. (255175)

42 reading.ti,ab. (118535)

43 (play or playing or playtime).ti,ab. (759787)

44 music.ti,ab. (17586)

45 boardgame*.ti,ab. (3)

46 games.ti,ab. (16559)

47 voluntary.ti,ab. (64481)

48 volunteering.ti,ab. (2125)

49 mentor*.ti,ab. (17791)

50 or/34-49 (7060335)

51 Homes for the Aged/ (14400)

52 Nursing Homes/ (36493)

53 care home*.ti,ab. (4517)

54 nursing home*.ti,ab. (31757)

55 residential care.ti,ab. (3647)

56 ((senior or elderly or old) adj day care).ti,ab. (46)

57 ((hospital* or ward) adj3 geriatric*).ti,ab. (3384)

58 community.ti,ab. (529188)

59 (sheltered adj (housing or accommodation)).ti,ab. (257)

60 (retirement adj (home* or village* or complex*)).ti,ab. (476)

61 (abbeyfield or almshouse*).ti,ab. (65)

62 (geriatric adj (institution* or care)).ti,ab. (2026)

63 assisted living.ti,ab. (2324)

64 own home.ti,ab. (1205)

65 (preschool or preschools).ti,ab. (26214)

66 playgroup*.ti,ab. (142)

67 (school or schools or college*).ti,ab. (398572)

68 (nursery or nurseries).ti,ab. (11824)

69 kindergarten*.ti,ab. (7051)

70 play setting*.ti,ab. (90)

71 (child care setting* or childcare setting*).ti,ab. (423)

72 (child* adj2 day care).ti,ab. (1110)

73 or/51-72 (987221)

74 8 and 18 and 33 and 50 (955)

75 8 and 18 and 50 and 73 (809)

76 8 and 33 and 50 and 73 (516)

77 7 or 74 or 75 or 76 (1567)

EMBASE via OvidSp

Database: Embase <1974 to 2021 July 26>

Search Strategy:

--------------------------------------------------------------------------------

1 (intergenerational or inter generational).ti,ab. (7195)

2 cross age.ti,ab. (116)

3 across generation*.ti,ab. (2274)

4 cross generation*.ti,ab. (503)

5 ((generations or different age groups or all ages or all age groups or mixed ages or mixed age groups or (old* adj2 young*)) adj5 (together or social engagement or connecting)).ti,ab. (315)

6 (intergenerational adj (program* or intervention*)).ti. (63)

7 or/1-5 (9973)

8 child*.ti,ab. (1839835)

9 (young adj (person or people or male* or female*)).ti,ab. (73378)

10 (youth* or teen*).ti,ab. (139345)

11 young offender*.ti,ab. (657)

12 (school and pupil*).ti,ab. (6479)

13 preschooler*.ti,ab. (8618)

14 student*.ti,ab. (398095)

15 (girl or girls or boy or boys).ti,ab. (319264)

16 or/8-15 (2474880)

17 dementia.ti,ab. (168681)

18 alzheimer*.ti,ab. (214580)

19 old*.ti,ab. (2218941)

20 elderly.ti,ab. (365236)

21 geriatric.ti,ab. (67443)

22 (residents or resident).ti,ab. (224475)

23 (elder or elders).ti,ab. (24475)

24 (retired or retiree*).ti,ab. (11390)

25 veteran*.ti,ab. (51001)

26 grandfriend*.ti,ab. (0)

27 seniors.ti,ab. (10783)

28 (senior adj citizen*).ti,ab. (1955)

29 (centarian* or centenarian* or nonagenarian* or octagenarian* or octogenarian* or sexagenarian* or septuagenarian*).ti,ab. (9976)

30 or/17-29 (2985765)

31 (Intergenerational adj5 program*).ti,ab. (252)

32 (Intergenerational adj5 activit*).ti,ab. (73)

33 interaction*.ti,ab. (1586512)

34 (Intergenerational adj5 (project or projects)).ti,ab. (51)

35 (intergenerational adj5 intervention*).ti,ab. (133)

36 initiative*.ti,ab. (135248)

37 scheme.ti,ab. (118860)

38 visit*.ti,ab. (411575)

39 reading.ti,ab. (137382)

40 (play or playing or playtime).ti,ab. (958236)

41 music.ti,ab. (21767)

42 boardgame*.ti,ab. (6)

43 games.ti,ab. (19999)

44 voluntary.ti,ab. (81898)

45 volunteering.ti,ab. (2684)

46 mentor*.ti,ab. (23424)

47 or/31-46 (3299598)

48 exp home for the aged/ (11191)

49 nursing home/ (55019)

50 care home*.ti,ab. (5719)

51 nursing home*.ti,ab. (40613)

52 residential care.ti,ab. (4537)

53 ((senior or elderly or old) adj day care).ti,ab. (51)

54 ((hospital* or ward) adj3 geriatric*).ti,ab. (5394)

55 community.ti,ab. (658292)

56 (sheltered adj (housing or accommodation)).ti,ab. (348)

57 (retirement adj (home* or village* or complex*)).ti,ab. (633)

58 (abbeyfield or almshouse*).ti,ab. (50)

59 (geriatric adj (institution* or care)).ti,ab. (2776)

60 assisted living.ti,ab. (3069)

61 own home.ti,ab. (1906)

62 (preschool or preschools).ti,ab. (30630)

63 playgroup*.ti,ab. (184)

64 (school or schools or college*).ti,ab. (574672)

65 (nursery or nurseries).ti,ab. (12133)

66 kindergarten*.ti,ab. (7954)

67 play setting*.ti,ab. (100)

68 (child care setting* or childcare setting*).ti,ab. (500)

69 (child* adj2 day care).ti,ab. (1243)

70 or/48-69 (1301852)

71 7 and 16 and 30 and 47 (382)

72 7 and 16 and 47 and 70 (411)

73 7 and 30 and 47 and 70 (270)

74 6 or 71 or 72 or 73 (707)

Psyc||NFO via OvidSp

Database: APA PsycInfo <1806 to July Week 2 2021>

Search Strategy:

--------------------------------------------------------------------------------

1 (intergenerational or inter generational).ti,ab. (10856)

2 cross age.ti,ab. (425)

3 across generation*.ti,ab. (1804)

4 cross generation*.ti,ab. (579)

5 ((generations or different age groups or all ages or all age groups or mixed ages or mixed age groups or (old* adj2 young*)) adj5 (together or social engagement or connecting)).ti,ab. (216)

6 (intergenerational adj2 (program* or intervention*)).ti. (183)

7 or/1-5 (13230)

8 child*.ti,ab. (718266)

9 (young adj (person or people or male* or female*)).ti,ab. (36091)

10 (youth* or teen*).ti,ab. (124134)

11 young offender*.ti,ab. (1382)

12 (school and pupil*).ti,ab. (12463)

13 preschooler*.ti,ab. (13210)

14 student*.ti,ab. (530181)

15 (girl or girls or boy or boys).ti,ab. (105641)

16 or/8-15 (1304003)

17 *retirement/ (3966)

18 *nursing home residents/ (1372)

19 dementia.ti,ab. (65851)

20 alzheimer*.ti,ab. (62559)

21 (old adj (adult* or people or aged)).ti,ab. (4118)

22 elderly.ti,ab. (58939)

23 geriatric.ti,ab. (14583)

24 (residents or resident).ti,ab. (47815)

25 (elder or elders).ti,ab. (10953)

26 (retired or retiree*).ti,ab. (4562)

27 veteran*.ti,ab. (22838)

28 pensioner*.ti,ab. (246)

29 grandfriend*.ti,ab. (2)

30 seniors.ti,ab. (7210)

31 (senior adj citizen*).ti,ab. (1063)

32 (centarian* or centenarian* or nonagenarian* or octagenarian* or octogenarian* or sexagenarian* or septuagenarian*).ti,ab. (740)

33 or/17-32 (242158)

34 (intergenerational adj10 program*).ti,ab. (610)

35 activit*.ti,ab. (447902)

36 interaction*.ti,ab. (326924)

37 (intergeneration adj10 (project or projects)).ti,ab. (0)

38 (intergenerational adj10 intervention*).ti,ab. (277)

39 initiative*.ti,ab. (44284)

40 scheme.ti,ab. (14753)

41 visit*.ti,ab. (55959)

42 reading.ti,ab. (115232)

43 (play or playing or playtime).ti,ab. (169593)

44 music.ti,ab. (31587)

45 boardgame*.ti,ab. (5)

46 games.ti,ab. (23001)

47 voluntary.ti,ab. (27777)

48 volunteering.ti,ab. (2920)

49 mentor*.ti,ab. (18031)

50 or/34-49 (1114650)

51 Nursing Homes/ (9029)

52 *retirement communities/ (282)

53 care home*.ti,ab. (1933)

54 nursing home*.ti,ab. (11930)

55 residential care.ti,ab. (3653)

56 ((senior or elderly or old) adj day care).ti,ab. (31)

57 ((hospital* or ward) adj3 geriatric*).ti,ab. (859)

58 (community and intergenerational).ti,ab. (1265)

59 (sheltered adj (housing or accommodation)).ti,ab. (134)

60 (retirement adj (home* or village* or complex*)).ti,ab. (270)

61 (abbeyfield or almshouse*).ti,ab. (43)

62 (geriatric adj (institution* or care)).ti,ab. (611)

63 assisted living.ti,ab. (1267)

64 own home.ti,ab. (633)

65 (preschool or preschools).ti,ab. (35382)

66 playgroup*.ti,ab. (241)

67 (school or schools or college*).ti,ab. (514342)

68 (nursery or nurseries).ti,ab. (4671)

69 kindergarten*.ti,ab. (18397)

70 play setting*.ti,ab. (294)

71 (child care setting* or childcare setting*).ti,ab. (538)

72 (child* adj2 day care).ti,ab. (437)

73 or/51-72 (571270)

74 7 and 16 and 33 and 50 (430)

75 7 and 16 and 50 and 73 (977)

76 7 and 33 and 50 and 73 (297)

77 6 or 74 or 75 or 76 (1307)

***************************

Database: Social Policy and Practice <202104>

Search Strategy:

--------------------------------------------------------------------------------

1 (intergenerational or inter generational).ti,ab. (1952)

2 cross age.ti,ab. (13)

3 across generation*.ti,ab. (105)

4 cross generation*.ti,ab. (33)

5 ((generations or different age groups or all ages or all age groups or mixed ages or mixed age groups or (old* adj2 young*)) adj5 (together or social engagement or connecting)).ti,ab. (96)

6 (intergenerational adj2 (program* or intervention*)).ti. (75)

7 or/1-5 (2077)

8 child*.ti,ab. (82146)

9 (young adj (person or people or male* or female*)).ti,ab. (25148)

10 (youth* or teen*).ti,ab. (13750)

11 young offender*.ti,ab. (1234)

12 (school and pupil*).ti,ab. (2759)

13 preschooler*.ti,ab. (99)

14 student*.ti,ab. (10175)

15 (girl or girls or boy or boys).ti,ab. (3953)

16 or/8-15 (109065)

17 dementia.ti,ab. (13307)

18 alzheimer*.ti,ab. (3134)

19 old*.ti,ab. (51433)

20 elderly.ti,ab. (14212)

21 geriatric.ti,ab. (2515)

22 (residents or resident).ti,ab. (11232)

23 (elder or elders).ti,ab. (3684)

24 (retired or retiree*).ti,ab. (819)

25 veteran*.ti,ab. (473)

26 grandfriend*.ti,ab. (1)

27 seniors.ti,ab. (461)

28 (senior adj citizen*).ti,ab. (196)

29 (centarian* or centenarian* or nonagenarian* or octagenarian* or octogenarian* or sexagenarian* or septuagenarian*).ti,ab. (180)

30 or/17-29 (80130)

31 program*.ti,ab. (34895)

32 activit*.ti,ab. (20836)

33 interaction*.ti,ab. (6648)

34 (project or projects).ti,ab. (26695)

35 intervention*.ti,ab. (27280)

36 initiative*.ti,ab. (15221)

37 scheme.ti,ab. (7787)

38 visit*.ti,ab. (5684)

39 reading.ti,ab. (1762)

40 (play or playing or playtime).ti,ab. (7707)

41 music.ti,ab. (944)

42 boardgame*.ti,ab. (0)

43 games.ti,ab. (790)

44 voluntary.ti,ab. (10402)

45 volunteering.ti,ab. (1431)

46 mentor*.ti,ab. (1383)

47 or/31-46 (125313)

48 care home*.ti,ab. (5287)

49 nursing home*.ti,ab. (4646)

50 residential care.ti,ab. (5649)

51 ((senior or elderly or old) adj day care).ti,ab. (7)

52 ((hospital* or ward) adj3 geriatric*).ti,ab. (236)

53 community.ti,ab. (49136)

54 (sheltered adj (housing or accommodation)).ti,ab. (1468)

55 (retirement adj (home* or village* or complex*)).ti,ab. (182)

56 (abbeyfield or almshouse*).ti,ab. (113)

57 (geriatric adj (institution* or care)).ti,ab. (167)

58 assisted living.ti,ab. (461)

59 own home.ti,ab. (455)

60 (preschool or preschools).ti,ab. (446)

61 playgroup*.ti,ab. (97)

62 (school or schools or college*).ti,ab. (24710)

63 (nursery or nurseries).ti,ab. (690)

64 kindergarten*.ti,ab. (93)

65 play setting*.ti,ab. (7)

66 (child care setting* or childcare setting*).ti,ab. (112)

67 (child* adj2 day care).ti,ab. (155)

68 or/48-67 (84371)

69 7 and 16 and 30 and 47 (325)

70 7 and 16 and 47 and 68 (259)

71 7 and 30 and 47 and 68 (285)

72 6 or 69 or 70 or 71 (538)

Database: HMIC Health Management Information Consortium <1979 to May 2021>

Search Strategy:

--------------------------------------------------------------------------------

1 (intergenerational or inter generational).ti,ab. (182)

2 cross age.ti,ab. (0)

3 across generation*.ti,ab. (37)

4 cross generation*.ti,ab. (11)

5 ((generations or different age groups or all ages or all age groups or mixed ages or mixed age groups or (old* adj2 young*)) adj5 (together or social engagement or connecting)).ti,ab. (10)

6 (intergenerational adj2 (program* or intervention*)).ti. (2)

7 or/1-5 (223)

8 child*.ti,ab. (31008)

9 (young adj (person or people or male* or female*)).ti,ab. (5636)

10 (youth* or teen*).ti,ab. (3627)

11 young offender*.ti,ab. (338)

12 (school and pupil*).ti,ab. (380)

13 preschooler*.ti,ab. (50)

14 student*.ti,ab. (6683)

15 (girl or girls or boy or boys).ti,ab. (1776)

16 or/8-15 (42746)

17 dementia.ti,ab. (3002)

18 alzheimer*.ti,ab. (601)

19 old*.ti,ab. (18096)

20 elderly.ti,ab. (8811)

21 geriatric.ti,ab. (1288)

22 (residents or resident).ti,ab. (4929)

23 (elder or elders).ti,ab. (564)

24 (retired or retiree*).ti,ab. (314)

25 veteran*.ti,ab. (469)

26 grandfriend*.ti,ab. (0)

27 seniors.ti,ab. (156)

28 (senior adj citizen*).ti,ab. (49)

29 (centarian* or centenarian* or nonagenarian* or octagenarian* or octogenarian* or sexagenarian* or septuagenarian*).ti,ab. (28)

30 or/17-29 (32364)

31 program*.ti,ab. (28482)

32 activit*.ti,ab. (15956)

33 interaction*.ti,ab. (4491)

34 (project or projects).ti,ab. (18310)

35 intervention*.ti,ab. (19407)

36 initiative*.ti,ab. (10545)

37 scheme.ti,ab. (6845)

38 visit*.ti,ab. (10874)

39 reading.ti,ab. (1249)

40 (play or playing or playtime).ti,ab. (4805)

41 music.ti,ab. (240)

42 boardgame*.ti,ab. (0)

43 games.ti,ab. (271)

44 voluntary.ti,ab. (6203)

45 volunteering.ti,ab. (279)

46 mentor*.ti,ab. (705)

47 or/31-46 (99116)

48 care home*.ti,ab. (1834)

49 nursing home*.ti,ab. (2158)

50 residential care.ti,ab. (2343)

51 ((senior or elderly or old) adj day care).ti,ab. (2)

52 ((hospital* or ward) adj3 geriatric*).ti,ab. (259)

53 community.ti,ab. (34580)

54 (sheltered adj (housing or accommodation)).ti,ab. (329)

55 (retirement adj (home* or village* or complex*)).ti,ab. (31)

56 (abbeyfield or almshouse*).ti,ab. (16)

57 (geriatric adj (institution* or care)).ti,ab. (99)

58 assisted living.ti,ab. (66)

59 own home.ti,ab. (187)

60 (preschool or preschools).ti,ab. (301)

61 playgroup*.ti,ab. (79)

62 (school or schools or college*).ti,ab. (12524)

63 (nursery or nurseries).ti,ab. (374)

64 kindergarten*.ti,ab. (59)

65 play setting*.ti,ab. (3)

66 (child care setting* or childcare setting*).ti,ab. (14)

67 (child* adj2 day care).ti,ab. (88)

68 or/48-67 (51037)

69 7 and 16 and 30 and 47 (8)

70 7 and 16 and 47 and 68 (9)

71 7 and 30 and 47 and 68 (6)

72 6 or 69 or 70 or 71 (18)

CINAHL via EBSCOhost

S135 S66 OR S132 OR S133 OR S134

S134 S67 and S92 and S109 and S131

S133 S67 and S77 and S109 and S131

S132 S67 AND S77 AND S92 AND S109

S131 S110 OR S111 OR S112 OR S113 OR S114 OR S115 OR S116 OR S117 OR S118 OR S119 OR S120 OR S121 OR S122 OR S123 OR S124 OR S125 OR S126 OR S127 OR S128 OR S129 OR S130

S130 TI ( (abbeyfield or almshouse*)) OR AB ( (abbeyfield or almshouse*))

S129 TI (child* N2 day care) OR AB (child* N2 day care)

S128 TI ( (child care setting* or childcare setting*)) OR AB ( (child care setting* or childcare setting*))

S127 TI play setting* OR AB play setting*

S126 TI kindergarten* OR AB kindergarten*

S125 TI ( (nursery or nurseries)) OR AB ( (nursery or nurseries))

S124 TI ( (school or schools or college*)) OR AB ( (school or schools or college*))

S123 TI playgroup* OR AB playgroup*

S122 TI ( (preschool or preschools)) OR AB ( (preschool or preschools))

S121 TI own home OR AB own home

S120 TI assisted living OR AB assisted living

S119 TI ( (geriatric N0 (institution* or care))) OR AB ( (geriatric N0 (institution* or care)))

S118 TI ( (retirement N0 (home* or village* or complex*))) OR AB ( (retirement N0 (home* or village* or complex*)))

S117 TI ( (sheltered N0 (housing or accommodation))) OR AB ( (sheltered N0 (housing or accommodation)))

S116 TI community OR AB community

S115 TI ( ((hospital* or ward) N3 geriatric*)) OR AB ( ((hospital* or ward) N3 geriatric*))

S114 TI ( ((senior or elderly or old) N0 day care)) OR AB ( ((senior or elderly or old) N0 day care))

S113 TI residential care OR AB residential care

S112 TI nursing home* OR AB nursing home*

S111 TI care home* OR AB care home*

S110 (MH “Nursing Homes”)

S109 S93 OR S94 OR S95 OR S96 OR S97 OR S98 OR S99 OR S100 OR S101 OR S102 OR S103 OR S104 OR S105 OR S106 OR S107 OR S108

S108 TI mentor* OR AB mentor*

S107 TI volunteering OR AB volunteering

S106 TI voluntary OR AB voluntary

S105 TI games OR AB games

S104 TI boardgame* OR AB boardgame*

S103 TI music OR AB music

S102 TI ( (play or playing or playtime)) OR AB ( (play or playing or playtime))

S101 TI reading OR AB reading

S100 TI visit* OR AB visit*

S99 TI scheme OR AB scheme

S98 TI initiative* OR AB initiative*

S97 TI intervention* OR AB intervention*

S96 TI ( (project or projects)) OR AB ( (project or projects))

S95 TI interaction* OR AB interaction*

Database - CINAHL Display

S94 TI activit* OR AB activit*

S93 TI program* OR AB program*

S92 S78 OR S79 OR S80 OR S81 OR S82 OR S83 OR S84 OR S85 OR S86 OR S87 OR S88 OR S89 OR S90 OR S91

S91 TI ( (centarian* or centenarian* or nonagenarian* or octagenarian* or octogenarian* or sexagenarian* or septuagenarian*)) AND AB ( (centarian* or centenarian* or nonagenarian* or octagenarian* or octogenarian* or sexagenarian* or septuagenarian*))

S90 TI (senior N0 citizen*) OR AB (senior N0 citizen*)

S89 TI seniors OR AB seniors

S88 TI grandfriend* OR AB grandfriend*

S87 TI veteran* OR AB veteran*

S86 TI ( (retired or retiree*)) OR AB ( (retired or retiree*))

S85 TI ( (elder or elders)) OR AB ( (elder or elders))

S84 TI ( (residents or resident)) OR AB ( (residents or resident))

S83 TI geriatric OR AB geriatric

S82 TI elderly OR AB elderly

S81 TI old* OR AB old*

S80 TI alzheimer* OR AB alzheimer*

S79 TI dementia OR AB dementia

S78 (MH “Aged”)

S77 S68 OR S69 OR S70 OR S71 OR S72 OR S73 OR S74 OR S75 OR S76

S76 TI ( (girl or girls or boy or boys)) OR AB ( (girl or girls or boy or boys))

S75 TI student* OR AB student*

S74 TI preschooler* OR AB preschooler*

S73 TI ( (school and pupil*)) OR AB ( (school and pupil*))

S72 TI young offender* OR AB young offender*

S71 TI ( (youth* or teen*)) OR AB ( (youth* or teen*))

S70 TI ( (young N0 (person or people or male* or female*))) OR AB ( (young N0 (person or people or male* or female*)))

S69 TI child* OR AB child*

S68 (MH “Adolescence”) OR (MH “Child”) OR (MH “Child, Preschool”)

S67 S60 OR S61 OR S62 OR S63 OR S64 OR S65

S66 TI (intergenerational N2 (program* or intervention*))

S65 TI ( ((generations or different age groups or all ages or all age groups or mixed ages or mixed age groups or (old* adj2 young*)) N5 (together or social engagement or connecting))) OR AB ( ((generations or different age groups or all ages or all age groups or mixed ages or mixed age groups or (old* adj2 young*)) N5 (together or social engagement or connecting)))

S64 TI cross generation* OR AB cross generation*

S63 TI across generation* OR AB across generation*

S62 TI cross age OR AB cross age

S61 TI ( (intergenerational or inter generational)) OR AB ( (intergenerational or inter generational))

S60 (MH “Intergenerational Relations”)

S59 TI ( (retirement N0 (home* or village* or complex*))) OR AB ( (retirement N0 (home* or village* or complex*)))

S58 TI ( (sheltered N0 (housing or accommodation))) OR AB ( (sheltered N0 (housing or accommodation)))

S57 TI community OR AB community

S56 TI ( ((hospital* or ward) N3 geriatric*)) OR AB ( ((hospital* or ward) N3 geriatric*))

S55 TI ( ((senior or elderly or old) N0 day care)) OR AB ( ((senior or elderly or old) N0 day care))

S54 TI residential care OR AB residential care

S53 TI nursing home* OR AB nursing home*

S52 TI care home* OR AB care home*

S51 (MH “Nursing Homes”)

S50 S34 OR S35 OR S36 OR S37 OR S38 OR S39 OR S40 OR S41 OR S42 OR S43 OR S44 OR S45 OR S46 OR S47 OR S48 OR S49

S49 TI mentor* OR AB mentor*

S48 TI volunteering OR AB volunteering

S47 TI voluntary OR AB voluntary

S46 TI games OR AB games

S45 TI boardgame* OR AB boardgame*

S44 TI music OR AB music

S43 TI ( (play or playing or playtime)) OR AB ( (play or playing or playtime))

S42 TI reading OR AB reading

S41 TI visit* OR AB visit*

S40 TI scheme OR AB scheme

S39 TI initiative* OR AB initiative*

S38 TI intervention* OR AB intervention*

S37 TI ( (project or projects)) OR AB ( (project or projects))

S36 TI interaction* OR AB interaction*

S35 TI activit* OR AB activit*

S34 TI program* OR AB program*

Database - CINAHL Display

S33 S19 OR S20 OR S21 OR S22 OR S23 OR S24 OR S25 OR S26 OR S27 OR S28 OR S29 OR S30 OR S31 OR S32

S32 TI ( (centarian* or centenarian* or nonagenarian* or octagenarian* or octogenarian* or sexagenarian* or septuagenarian*)) AND AB ( (centarian* or centenarian* or nonagenarian* or octagenarian* or octogenarian* or sexagenarian* or septuagenarian*))

S31 TI (senior N0 citizen*) OR AB (senior N0 citizen*)

S30 TI seniors OR AB seniors

S29 TI grandfriend* OR AB grandfriend*

S28 TI veteran* OR AB veteran*

S27 TI ( (retired or retiree*)) OR AB ( (retired or retiree*))

S26 TI ( (elder or elders)) OR AB ( (elder or elders))

S25 TI ( (residents or resident)) OR AB ( (residents or resident))

S24 TI geriatric OR AB geriatric

S23 TI elderly OR AB elderly

S22 TI old* OR AB old*

S21 TI alzheimer* OR AB alzheimer*

S20 TI dementia OR AB dementia

S19 (MH “Aged”)

S18 S9 OR S10 OR S11 OR S12 OR S13 OR S14 OR S15 OR S16 OR S17

S17 TI ( (girl or girls or boy or boys)) OR AB ( (girl or girls or boy or boys))

S16 TI student* OR AB student*

S15 TI preschooler* OR AB preschooler*

S14 TI ( (school and pupil*)) OR AB ( (school and pupil*))

S13 TI young offender* OR AB young offender*

S12 TI ( (youth* or teen*)) OR AB ( (youth* or teen*))

S11 TI ( (young N0 (person or people or male* or female*))) OR AB ( (young N0 (person or people or male* or female*)))

S10 TI child* OR AB child*

S9 (MH “Adolescence”) OR (MH “Child”) OR (MH “Child, Preschool”)

S8 S1 OR S2 OR S3 OR S4 OR S5 OR S6

S7 TI (intergenerational N2 (program* or intervention*))

S6 TI ( ((generations or different age groups or all ages or all age groups or mixed ages or mixed age groups or (old* adj2 young*)) N5 (together or social engagement or connecting))) OR AB ( ((generations or different age groups or all ages or all age groups or mixed ages or mixed age groups or (old* adj2 young*)) N5 (together or social engagement or connecting)))

S5 TI cross generation* OR AB cross generation*

S4 TI across generation* OR AB across generation*

S3 TI cross age OR AB cross age

S2 TI ( (intergenerational or inter generational)) OR AB ( (intergenerational or inter generational))

S1 (MH “Intergenerational Relations”)

AgeLine via EBSCOhost

S5 S3 OR S4

S4 TI ( (volunteer* or “voluntary”) W5 (school* or playgroup* or “play setting*“ or kindergarten*

or nurser*)) OR AB ( (volunteer* or “voluntary”) W5 (school* or playgroup* or “play setting*“ or kindergarten* or nurser*))

S3 S1 OR S2

S2 TI ( intergenerational W1 (program* or intervention* or project* or initiative* or scheme*)) OR AB ( intergenerational W1 (program* or intervention* or project* or initiative* or scheme*))

S1 DE “Intergenerational Programs”

ERIC via EBSCOhost

Search Terms Search Options

S4 S1 OR S2 OR S3

S3 TI intergenerational

S2 AB ( TI ( (volunteer* or “voluntary”) W5 (“old aged” or elderly or “geriatric” or pensioner* or veteran* or older))) OR TI ( TI ( (volunteer* or “voluntary”) W5 (“old aged” or elderly or “geriatric” or pensioner* or veteran* or older)))

S1 TI ( intergenerational W1 (program* or intervention* or project* or initiative* or scheme*)) OR AB ( intergenerational W1 (program* or intervention* or project* or initiative* or scheme*))

ASSIA via ProQuest

(intergenerational NEAR/2 (program* OR intervention*)) OR ((MAINSUBJECT.EXACT(“Intergenerational relationships”) OR (ti((intergenerational OR inter generational)) OR ab((intergenerational OR inter generational))) OR (ti(cross age) OR ab(cross age)) OR (ti(across generation*) OR ab(across generation*)) OR (ti(cross generation*) OR ab(cross generation*)) OR (((ti((generations OR different age groups OR all ages OR all age groups OR mixed ages OR mixed age groups)) OR ab((generations OR different age groups OR all ages OR all age groups OR mixed ages OR mixed age groups))) OR (ti((“old* and young*“ OR “old* people and young* people” OR “old* persons and young* persons” OR “old* generation* and young* generation*“)) OR ab((“old* and young*“ OR “old* people and young* people” OR “old* persons and young* persons” OR “old* generation* and young* generation*“)))) AND (ti((together OR social engagement OR connecting)) OR ab((together OR social engagement OR connecting))))) AND ((ti(child*) OR ab(child*)) OR (ti((young NEAR/0 (person OR people OR male* OR female*))) OR ab((young NEAR/0 (person OR people OR male* OR female*)))) OR

(ti((youth* OR teen*)) OR ab((youth* OR teen*))) OR (ti(young offender*) OR ab(young offender*)) OR (ti((school AND pupil*)) OR ab((school AND pupil*))) OR (ti(preschooler*) OR ab(preschooler*)) OR (ti(student*) OR ab(student*)) OR (ti((girl OR girls OR boy OR boys)) OR ab((girl OR girls OR boy OR boys)))) AND (MAINSUBJECT.EXACT(“Elderly people”) OR (ti(dementia) OR ab(dementia)) OR (ti(alzheimer*) OR ab(alzheimer*)) OR (ti(old*) OR ab(old*)) OR (ti(elderly) OR ab(elderly)) OR (ti(geriatric) OR ab(geriatric)) OR (ti((residents OR resident)) OR ab((residents OR resident))) OR (ti((elder OR elders)) OR ab((elder OR elders))) OR (ti((retired OR retiree*)) OR ab((retired OR retiree*))) OR (ti(veteran*) OR ab(veteran*)) OR (ti(grandfriend*) OR ab(grandfriend*)) OR grandfriend* OR (ti(seniors) OR ab(seniors)) OR (ti((senior NEAR/0 citizen*)) OR ab((senior NEAR/0 citizen*))) OR (ti((centarian* OR centenarian* OR nonagenarian* OR octagenarian* OR octogenarian* OR sexagenarian* OR septuagenarian*)) OR ab((centarian* OR centenarian* OR nonagenarian* OR octagenarian* OR octogenarian* OR sexagenarian* OR septuagenarian*)))) AND ((ti(intergeneration* NEAR/10 program*) OR ab(intergeneration* NEAR/10 program*)) OR (ti(activit*) OR ab(activit*)) OR (ti(interaction*) OR ab(interaction*)) OR (ti((intergeneration* NEAR/10 project OR intergeneration* NEAR/10 projects)) OR ab((intergeneration* NEAR/10 project OR intergeneration* NEAR/10 projects))) OR (ti(intergeneration* NEAR/10 intervention*) OR ab(intergeneration* NEAR/10 intervention*)) OR (ti(initiative*) OR ab(initiative*)) OR (ti(scheme) OR ab(scheme)) OR (ti(visit*) OR ab(visit*)) OR (ti(reading) OR ab(reading)) OR (ti((play OR playing OR playtime)) OR ab((play OR playing OR playtime))) OR (ti(music) OR ab(music)) OR (ti(boardgame*) OR ab(boardgame*)) OR (ti(games) OR ab(games)) OR (ti(volunteering) OR ab(volunteering)) OR (ti(mentor*) OR ab(mentor*)))) OR ((MAINSUBJECT.EXACT(“Intergenerational relationships”) OR (ti((intergenerational OR inter generational)) OR ab((intergenerational OR inter generational))) OR (ti(cross age) OR ab(cross age)) OR (ti(across generation*) OR ab(across generation*)) OR (ti(cross generation*) OR ab(cross generation*)) OR (((ti((generations OR different age groups OR all ages OR all age groups OR mixed ages OR mixed age groups)) OR ab((generations OR different age groups OR all ages OR all age groups OR mixed ages OR mixed age groups))) OR (ti((“old* and young*“ OR “old* people and young* people” OR “old* persons and young* persons” OR “old* generation* and young* generation*“)) OR ab((“old* and young*“ OR “old* people and young* people” OR “old* persons and young* persons” OR “old* generation* and young* generation*“)))) AND (ti((together OR social engagement OR connecting)) OR ab((together OR social engagement OR connecting))))) AND ((ti(child*) OR ab(child*)) OR (ti((young NEAR/0 (person OR people OR male* OR female*))) OR ab((young NEAR/0 (person OR people OR male* OR female*)))) OR (ti((youth* OR teen*)) OR ab((youth* OR teen*))) OR (ti(young offender*) OR ab(young offender*)) OR (ti((school AND pupil*)) OR ab((school AND pupil*))) OR (ti(preschooler*) OR ab(preschooler*)) OR (ti(student*) OR ab(student*)) OR (ti((girl OR girls OR boy OR boys)) OR ab((girl OR girls OR boy OR boys)))) AND ((ti(intergeneration* NEAR/10 program*) OR

ab(intergeneration* NEAR/10 program*)) OR (ti(activit*) OR ab(activit*)) OR (ti(interaction*) OR ab(interaction*)) OR (ti((intergeneration* NEAR/10 project OR intergeneration* NEAR/10 projects)) OR ab((intergeneration* NEAR/10 project OR intergeneration* NEAR/10 projects))) OR (ti(intergeneration* NEAR/10 intervention*) OR ab(intergeneration* NEAR/10 intervention*)) OR (ti(initiative*) OR ab(initiative*)) OR (ti(scheme) OR ab(scheme)) OR (ti(visit*) OR ab(visit*)) OR (ti(reading) OR ab(reading)) OR (ti((play OR playing OR playtime)) OR ab((play OR playing OR playtime))) OR (ti(music) OR ab(music)) OR (ti(boardgame*) OR ab(boardgame*)) OR (ti(games) OR ab(games)) OR (ti(volunteering) OR ab(volunteering)) OR (ti(mentor*) OR ab(mentor*))) AND ((MAINSUBJECT.EXACT(“Nursing homes”) OR MAINSUBJECT.EXACT(“Private nursing homes”)) OR (ti(care home*) OR ab(care home*)) OR (ti(nursing home*) OR ab(nursing home*)) OR (ti(residential care) OR ab(residential care)) OR (ti(((senior OR elderly OR old) NEAR/0 day care)) OR ab(((senior OR elderly OR old) NEAR/0 day care))) OR (ti(((hospital* OR ward) NEAR/3 geriatric*)) OR ab(((hospital* OR ward) NEAR/3 geriatric*))) OR (ti(community) OR ab(community)) OR (ti((sheltered NEAR/0 (housing OR accommodation))) OR ab((sheltered NEAR/0 (housing OR accommodation)))) OR (ti((retirement adj (home* OR village* OR complex*))) OR ab((retirement adj (home* OR village* OR complex*)))) OR (ti((retirement NEAR/0 (home* OR village* OR complex*))) OR ab((retirement NEAR/0 (home* OR village* OR complex*)))) OR (ti((abbeyfield OR almshouse*)) OR ab((abbeyfield OR almshouse*))) OR (ti((geriatric NEAR/0 (institution* OR care))) OR ab((geriatric NEAR/0 (institution* OR care)))) OR (ti(assisted living) OR ab(assisted living)) OR (ti(own home) OR ab(own home)) OR (ti((preschool OR preschools)) OR ab((preschool OR preschools))) OR (ti(playgroup*) OR ab(playgroup*)) OR (ti((school OR schools OR college*)) OR ab((school OR schools OR college*))) OR (ti((nursery OR nurseries)) OR ab((nursery OR nurseries))) OR (ti(kindergarten*) OR ab(kindergarten*)) OR (ti(play setting*) OR ab(play setting*)) OR (ti((child care setting* OR childcare setting*)) OR ab((child care setting* OR childcare setting*))) OR (ti((child* NEAR/2 day care)) OR ab((child* NEAR/2 day care))))) OR ((MAINSUBJECT.EXACT(“Intergenerational relationships”) OR (ti((intergenerational OR inter generational)) OR ab((intergenerational OR inter generational))) OR (ti(cross age) OR ab(cross age)) OR (ti(across generation*) OR ab(across generation*)) OR (ti(cross generation*) OR ab(cross generation*)) OR (((ti((generations OR different age groups OR all ages OR all age groups OR mixed ages OR mixed age groups)) OR ab((generations OR different age groups OR all ages OR all age groups OR mixed ages OR mixed age groups))) OR (ti((“old* and young*“ OR “old* people and young* people” OR “old* persons and young* persons” OR “old* generation* and young* generation*“)) OR ab((“old* and young*“ OR “old* people and young* people” OR “old* persons and young* persons” OR “old* generation* and young* generation*“)))) AND (ti((together OR social engagement OR connecting)) OR ab((together OR social engagement OR connecting))))) AND (MAINSUBJECT.EXACT(“Elderly people”) OR (ti(dementia) OR ab(dementia)) OR (ti(alzheimer*) OR ab(alzheimer*)) OR (ti(old*) OR ab(old*))

OR (ti(elderly) OR ab(elderly)) OR (ti(geriatric) OR ab(geriatric)) OR (ti((residents OR resident)) OR ab((residents OR resident))) OR (ti((elder OR elders)) OR ab((elder OR elders))) OR (ti((retired OR retiree*)) OR ab((retired OR retiree*))) OR (ti(veteran*) OR ab(veteran*)) OR (ti(grandfriend*) OR ab(grandfriend*)) OR grandfriend* OR (ti(seniors) OR ab(seniors)) OR (ti((senior NEAR/0 citizen*)) OR ab((senior NEAR/0 citizen*))) OR (ti((centarian* OR centenarian* OR nonagenarian* OR octagenarian* OR octogenarian* OR sexagenarian* OR septuagenarian*)) OR ab((centarian* OR centenarian* OR nonagenarian* OR octagenarian* OR octogenarian* OR sexagenarian* OR septuagenarian*)))) AND ((ti(intergeneration* NEAR/10 program*) OR ab(intergeneration* NEAR/10 program*)) OR (ti(activit*) OR ab(activit*)) OR (ti(interaction*) OR ab(interaction*)) OR (ti((intergeneration* NEAR/10 project OR intergeneration* NEAR/10 projects)) OR ab((intergeneration* NEAR/10 project OR intergeneration* NEAR/10 projects))) OR (ti(intergeneration* NEAR/10 intervention*) OR ab(intergeneration* NEAR/10 intervention*)) OR (ti(initiative*) OR ab(initiative*)) OR (ti(scheme) OR ab(scheme)) OR (ti(visit*) OR ab(visit*)) OR (ti(reading) OR ab(reading)) OR (ti((play OR playing OR playtime)) OR ab((play OR playing OR playtime))) OR (ti(music) OR ab(music)) OR (ti(boardgame*) OR ab(boardgame*)) OR (ti(games) OR ab(games)) OR (ti(volunteering) OR ab(volunteering)) OR (ti(mentor*) OR ab(mentor*))) AND ((MAINSUBJECT.EXACT(“Nursing homes”) OR MAINSUBJECT.EXACT(“Private nursing homes”)) OR (ti(care home*) OR ab(care home*)) OR (ti(nursing home*) OR ab(nursing home*)) OR (ti(residential care) OR ab(residential care)) OR (ti(((senior OR elderly OR old) NEAR/0 day care)) OR ab(((senior OR elderly OR old) NEAR/0 day care))) OR (ti(((hospital* OR ward) NEAR/3 geriatric*)) OR ab(((hospital* OR ward) NEAR/3 geriatric*))) OR (ti(community) OR ab(community)) OR (ti((sheltered NEAR/0 (housing OR accommodation))) OR ab((sheltered NEAR/0 (housing OR accommodation)))) OR (ti((retirement adj (home* OR village* OR complex*))) OR ab((retirement adj (home* OR village* OR complex*)))) OR (ti((retirement NEAR/0 (home* OR village* OR complex*))) OR ab((retirement NEAR/0 (home* OR village* OR complex*)))) OR (ti((abbeyfield OR almshouse*)) OR ab((abbeyfield OR almshouse*))) OR (ti((geriatric NEAR/0 (institution* OR care))) OR ab((geriatric NEAR/0 (institution* OR care)))) OR (ti(assisted living) OR ab(assisted living)) OR (ti(own home) OR ab(own home)) OR (ti((preschool OR preschools)) OR ab((preschool OR preschools))) OR (ti(playgroup*) OR ab(playgroup*)) OR (ti((school OR schools OR college*)) OR ab((school OR schools OR college*))) OR (ti((nursery OR nurseries)) OR ab((nursery OR nurseries))) OR (ti(kindergarten*) OR ab(kindergarten*)) OR (ti(play setting*) OR ab(play setting*)) OR (ti((child care setting* OR childcare setting*)) OR ab((child care setting* OR childcare setting*))) OR (ti((child* NEAR/2 day care)) OR ab((child* NEAR/2 day care)))))

ProQuest Dissertations and Theses

ti((intergenerational NEAR/2 (program* OR intervention*))) OR ((ti((intergenerational OR inter generational)) OR ti(cross age) OR ti(cross generation*) OR ti((generations OR different age groups OR all ages OR all age groups OR mixed ages OR mixed age groups))) AND ((ti(child*) OR ab(child*)) OR (ti((young NEAR/0 (person OR people OR male* OR female*))) OR ab((young NEAR/0 (person OR people OR male* OR female*)))) OR (ti((youth* OR teen*)) OR ab((youth* OR teen*))) OR (ti(young offender*) OR ab(young offender*)) OR (ti((school AND pupil*)) OR ab((school AND pupil*))) OR (ti(preschooler*) OR ab(preschooler*)) OR (ti(student*) OR ab(student*)) OR (ti((girl OR girls OR boy OR boys)) OR ab((girl OR girls OR boy OR boys)))) AND ((ti(dementia) OR ab(dementia)) OR (ti(alzheimer) OR ab(alzheimer)) OR (ti(old*) OR ab(old*)) OR (ti(elderly) OR ab(elderly)) OR (ti(geriatric) OR ab(geriatric)) OR (ti((residents OR resident)) OR ab((residents OR resident))) OR (ti((elder OR elders)) OR ab((elder OR elders))) OR (ti((retired OR retiree*)) OR ab((retired OR retiree*))) OR (ti(veteran*) OR ab(veteran*)) OR (ti(grandfriend*) OR ab(grandfriend*)) OR (ti(seniors) OR ab(seniors)) OR (ti((senior NEAR/0 citizen*)) OR ab((senior NEAR/0 citizen*))) OR (ti((centarian* OR centenarian* OR nonagenarian* OR octagenarian* OR octogenarian* OR sexagenarian* OR septuagenarian*)) OR ab((centarian* OR centenarian* OR nonagenarian* OR octagenarian* OR octogenarian* OR sexagenarian* OR septuagenarian*)))) AND ((ti(intergenerational NEAR/10 program*) OR ab(intergenerational NEAR/10 program*)) OR (ti(activit*) OR ab(activit*)) OR (ti(interaction*) OR ab(interaction*)) OR (ti(intergenerational NEAR/10 (project OR projects)) OR ab(intergenerational NEAR/10 (project OR projects))) OR (ti(intergenerational NEAR/10 intervention*) OR ab(intergenerational NEAR/10 intervention*)) OR (ti(initiative*) OR ab(initiative*)) OR (ti(scheme) OR ab(scheme)) OR (ti(visit*) OR ab(visit*)) OR (ti(reading) OR ab(reading)) OR (ti((play OR playing OR playtime)) OR ab((play OR playing OR playtime))) OR (ti(music) OR ab(music)) OR (ti(boardgame*) OR ab(boardgame*)) OR (ti(games) OR ab(games)) OR (ti(voluntary) OR ab(voluntary)) OR (ti(volunteering) OR ab(volunteering)) OR (ti(mentor*) OR ab(mentor*)))) OR ((ti((intergenerational OR inter generational)) OR ti(cross age) OR ti(cross generation*) OR ti((generations OR different age groups OR all ages OR all age groups OR mixed ages OR mixed age groups))) AND ((ti(child*) OR ab(child*)) OR (ti((young NEAR/0 (person OR people OR male* OR female*))) OR ab((young NEAR/0 (person OR people OR male* OR female*)))) OR (ti((youth* OR teen*)) OR ab((youth* OR teen*))) OR (ti(young offender*) OR ab(young offender*)) OR (ti((school AND pupil*)) OR ab((school AND pupil*))) OR (ti(preschooler*) OR ab(preschooler*)) OR (ti(student*) OR ab(student*)) OR (ti((girl OR girls OR boy OR boys)) OR ab((girl OR girls OR boy OR boys)))) AND ((ti(intergenerational NEAR/10 program*) OR ab(intergenerational NEAR/10 program*)) OR (ti(activit*) OR ab(activit*)) OR (ti(interaction*) OR ab(interaction*)) OR (ti(intergenerational NEAR/10 (project OR projects)) OR ab(intergenerational NEAR/10 (project OR projects))) OR (ti(intergenerational NEAR/10 intervention*) OR ab(intergenerational NEAR/10 intervention*)) OR (ti(initiative*) OR ab(initiative*)) OR (ti(scheme) OR ab(scheme)) OR (ti(visit*)

OR ab(visit*)) OR (ti(reading) OR ab(reading)) OR (ti((play OR playing OR playtime)) OR ab((play OR playing OR playtime))) OR (ti(music) OR ab(music)) OR (ti(boardgame*) OR ab(boardgame*)) OR (ti(games) OR ab(games)) OR (ti(voluntary) OR ab(voluntary)) OR (ti(volunteering) OR ab(volunteering)) OR (ti(mentor*) OR ab(mentor*))) AND ((ti(care home*) OR ab(care home*)) OR (ti(nursing home*) OR ab(nursing home*)) OR (ti(residential care) OR ab(residential care)) OR (ti(((senior OR elderly OR old) NEAR/0 day care)) OR ab(((senior OR elderly OR old) NEAR/0 day care))) OR (ti(((hospital* OR ward) NEAR/3 geriatric*)) OR ab(((hospital* OR ward) NEAR/3 geriatric*))) OR (ti(community) OR ab(community)) OR (ti((sheltered NEAR/0 (housing OR accommodation))) OR ab((sheltered NEAR/0 (housing OR accommodation)))) OR (ti((retirement NEAR/0 (home* OR village* OR complex*))) OR ab((retirement NEAR/0 (home* OR village* OR complex*)))) OR (ti((abbeyfield OR almshouse*)) OR ab((abbeyfield OR almshouse*))) OR (ti((geriatric NEAR/0 (institution* OR care))) OR ab((geriatric NEAR/0 (institution* OR care)))) OR (ti(assisted living) OR ab(assisted living)) OR (ti(own home) OR ab(own home)) OR (ti((preschool OR preschools)) OR ab((preschool OR preschools))) OR (ti(playgroup*) OR ab(playgroup*)) OR (ti((school OR schools OR college*)) OR ab((school OR schools OR college*))) OR (ti((nursery OR nurseries)) OR ab((nursery OR nurseries))) OR (ti(kindergarten*) OR ab(kindergarten*)) OR (ti(play setting*) OR ab(play setting*)) OR (ti((child* NEAR/2 day care)) OR ab((child* NEAR/2 day care))))) OR ((ti((intergenerational OR inter generational)) OR ti(cross age) OR ti(cross generation*) OR ti((generations OR different age groups OR all ages OR all age groups OR mixed ages OR mixed age groups))) AND ((ti(dementia) OR ab(dementia)) OR (ti(alzheimer) OR ab(alzheimer)) OR (ti(old*) OR ab(old*)) OR (ti(elderly) OR ab(elderly)) OR (ti(geriatric) OR ab(geriatric)) OR (ti((residents OR resident)) OR ab((residents OR resident))) OR (ti((elder OR elders)) OR ab((elder OR elders))) OR (ti((retired OR retiree*)) OR ab((retired OR retiree*))) OR (ti(veteran*) OR ab(veteran*)) OR (ti(grandfriend*) OR ab(grandfriend*)) OR (ti(seniors) OR ab(seniors)) OR (ti((senior NEAR/0 citizen*)) OR ab((senior NEAR/0 citizen*))) OR (ti((centarian* OR centenarian* OR nonagenarian* OR octagenarian* OR octogenarian* OR sexagenarian* OR septuagenarian*)) OR ab((centarian* OR centenarian* OR nonagenarian* OR octagenarian* OR octogenarian* OR sexagenarian* OR septuagenarian*)))) AND ((ti(intergenerational NEAR/10 program*) OR ab(intergenerational NEAR/10 program*)) OR (ti(activit*) OR ab(activit*)) OR (ti(interaction*) OR ab(interaction*)) OR (ti(intergenerational NEAR/10 (project OR projects)) OR ab(intergenerational NEAR/10 (project OR projects))) OR (ti(intergenerational NEAR/10 intervention*) OR ab(intergenerational NEAR/10 intervention*)) OR (ti(initiative*) OR ab(initiative*)) OR (ti(scheme) OR ab(scheme)) OR (ti(visit*) OR ab(visit*)) OR (ti(reading) OR ab(reading)) OR (ti((play OR playing OR playtime)) OR ab((play OR playing OR playtime))) OR (ti(music) OR ab(music)) OR (ti(boardgame*) OR ab(boardgame*)) OR (ti(games) OR ab(games)) OR (ti(voluntary) OR ab(voluntary)) OR (ti(volunteering) OR ab(volunteering)) OR (ti(mentor*) OR ab(mentor*))) AND

((ti(care home*) OR ab(care home*)) OR (ti(nursing home*) OR ab(nursing home*)) OR (ti(residential care) OR ab(residential care)) OR (ti(((senior OR elderly OR old) NEAR/0 day care)) OR ab(((senior OR elderly OR old) NEAR/0 day care))) OR (ti(((hospital* OR ward) NEAR/3 geriatric*)) OR ab(((hospital* OR ward) NEAR/3 geriatric*))) OR (ti(community) OR ab(community)) OR (ti((sheltered NEAR/0 (housing OR accommodation))) OR ab((sheltered NEAR/0 (housing OR accommodation)))) OR (ti((retirement NEAR/0 (home* OR village* OR complex*))) OR ab((retirement NEAR/0 (home* OR village* OR complex*)))) OR (ti((abbeyfield OR almshouse*)) OR ab((abbeyfield OR almshouse*))) OR (ti((geriatric NEAR/0 (institution* OR care))) OR ab((geriatric NEAR/0 (institution* OR care)))) OR (ti(assisted living) OR ab(assisted living)) OR (ti(own home) OR ab(own home)) OR (ti((preschool OR preschools)) OR ab((preschool OR preschools))) OR (ti(playgroup*) OR ab(playgroup*)) OR (ti((school OR schools OR college*)) OR ab((school OR schools OR college*))) OR (ti((nursery OR nurseries)) OR ab((nursery OR nurseries))) OR (ti(kindergarten*) OR ab(kindergarten*)) OR (ti(play setting*) OR ab(play setting*)) OR (ti((child* NEAR/2 day care)) OR ab((child* NEAR/2 day care)))))

SSCI and CPSI-S via Web of Science

#1 or #2 or #3 or #4............

## 2 Appendix B - Progress Plus reporting

[Enter text here]

| Study | Residence | Race/ethnicity/culture | Occupation | Gender/sex | Religion | Education | Socioeconomic status | Social capital | Other personal characteristics | Relationship features | Time dependent relationship features |
| --- | --- | --- | --- | --- | --- | --- | --- | --- | --- | --- | --- |
| Carcavilla (2020) |  | OP Spanish  CYP Italian |  | 61% F  CYP 100% F |  | CYP in secondary school |  |  | Cognitive impairment excluded |  |  |
| Cardona (2002) |  |  |  | 66.6% F  33.3% M |  |  |  |  | Cognitive impairment excluded |  |  |
| Chippendale (2015) |  | White 57.5%  African american 36% Middle eastern 12.5% |  |  |  |  |  |  |  |  |  |
| Dawson (2017) |  | White 73% Black/African American 20%  Other 6% |  | 60% F  40% M |  | All had completed high school |  |  |  |  |  |
| Detmer (2020) |  |  |  |  |  |  |  |  | Cognitive impairment  Physical health impairments |  |  |
| George (2011) |  |  |  | 13 F  2 M |  |  |  |  | Cognitive impairment |  |  |
| Giglio (2006) |  |  |  | 5 F  12 M |  |  |  |  | Cognitive impairment |  |  |
| Gruenewald (2016) |  | Overall sample  92% black/african american 5% white/caucasian 3% other 85% female |  |  |  | Overall 44% less than or equal to high school achievement | Overall 30% earned less than $15000 |  | Cognitive impairment excluded  Physical health impairments |  |  |
| Low (2015) |  |  |  | 80% F |  |  |  |  | Cognitive impairment |  |  |
| Rook (2003) |  | Caucasian 90% |  | 65.6% F |  |  | Low SES less than $15000 income (69%) |  | Child physical health impairments |  |  |
| Sakurai (2018) |  |  |  | 82.4% F |  |  |  |  | Physical health impairments |  |  |
| Shkilnyk (1984) |  |  |  |  |  |  |  |  | Physical health impairments - 13 elders relatively mobile and in good health  16 elders limited mobility and poor state of health |  |  |
| Sipsas-Herrmann (2000) |  | Child multi-ethnic sample  80% anglo  15% hispanic  5% other |  | 102 F  92 M |  |  | Middle to high SES |  |  |  |  |
| Thornton (2018) |  | White non-hispanic 70%  Hispanic 10%  African american 11% American indian 7% Asian 5%  CYP 33% white non-hispanic |  | 81% F  19% M |  | 79% at least college or vocational training  CYP all completed high school or higher | 72% annual income less than 20,000$  CYP 19% less than $20,000 income  61% had more than $40,000 income |  |  |  |  |
